# A concise synthesis of 3-(1-alkenyl)isoindolin-1-ones and 5-(1-alkenyl)pyrrol-2-ones by the intermolecular coupling reactions of *N*-acyliminium ions with unactivated olefins

**DOI:** 10.3762/bjoc.8.21

**Published:** 2012-02-06

**Authors:** Nianhong Lu, Lihong Wang, Zhanshan Li, Wei Zhang

**Affiliations:** 1 State Key Laboratory of Applied Organic Chemistry, Lanzhou University, Lanzhou 730000, China; Fax: +86 (931) 8625657

**Keywords:** 3-(1-alkenyl)isoindol-1-ones, 5-(1-alkenyl)pyrrol-2-ones, coupling reaction, 2,3-dihydro-3-hydroxyisoindol-1-one, 2,5-dihydro-5-hydroxypyrrol-2-one, *N*-acyliminium ions

## Abstract

A concise synthesis of 3-(1-alkenyl)isoindolin-1-ones and 5-(1-alkenyl)pyrrol-2-ones has been achieved by the coupling reactions of *N*-acyliminium ions produced from 3-hydroxyisoindol-1-ones or 5-hydroxy-1-pyrrol-2-ones with unactivated olefins in the presence of BF_3_·OEt_2_ at room temperature. For most of the olefins, the reactions afforded the C_sp3_–C_sp2_ cross-coupling products, but for the α-methylstyrene and 1-hexene, the C_sp3_–C_sp3_ cross-coupling products were obtained.

## Introduction

**Scheme 1:**
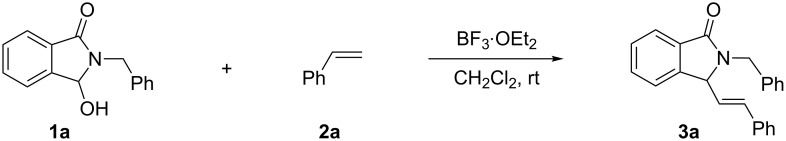
Reaction of 3-hydroxyisoindol-1-one with styrene.

**Scheme 2:**
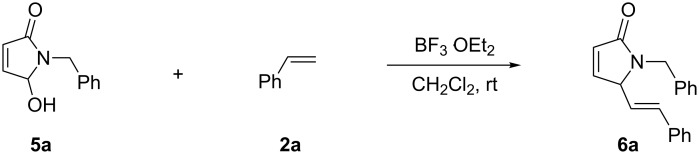
Reaction of 5-hydroxypyrrol-1-one with styrene.

The coupling of alcohols with alkynes, aromatics and active methylene compounds has attracted great attention in recent years as an effective and environmentally benign strategy for the construction of carbon–carbon bonds with the concomitant loss of water. For example, the metal-catalyzed coupling of allyl, benzyl, and propargyl alcohols with terminal alkynes to give the doubly alkyl-substituted acetylenes [1–3]; the Brønsted acid and Lewis acid catalyzed coupling of alcohols with indoles to give the 3-alkyl-substituted indoles [4–6]; and the Brønsted acid and Lewis acid-catalyzed coupling of alcohols with 1,3-dicarbonyls to give the 2-alkyl-substituted 1,3-dicarbonyls [7–9]. All these reactions generally proceed by the addition of carbon cations to multiple bonds and subsequent deprotonation. In comparison, the reports for the coupling of alcohols with unactivated olefins to give the corresponding alkyl-substituted alkenes are rare. Lee recently reported a coupling of alcohols with olefins catalyzed by a ruthenium complex to give alkyl-substituted alkenes through the formation of C_sp3_–C_sp2_ bonds [10]; Liu reported the FeCl_3_/TsOH catalyzed coupling of diarylmethanol with styrenes to afford the alkyl-substituted styrenes [11]. We have long been interested in the reactions of *N*-acyliminium ions produced easily by the Brønsted acid and Lewis acid catalyzed dehydroxylation of α-hydroxyamides [12–14]. The high electrophilicity of these species is very suitable for electrophilic addition to carbon–carbon multiple bonds. The coupling reactions of *N*-acyliminium ions with various carbon nucleophiles, such as allylsilanes, alkylmetals, TMSCN, 1,3-dicarbonyls, isonitriles, enol derivatives and aromatics has been studied extensively [15–17]. Few reports are found to deal with the intermolecular coupling reactions of *N*-acyliminium ions with unactivated olefins, although the intramolecular addition of acyliminium ions to olefins has been reported [18]. The reported olefins that coupled with *N*-acyliminium ions were generally activated alkenes, such as 1-alkenylsilanes [19], 1-alkenylcoppers [20–21], 1-alkenylalanes [22] and 1-alkenylboronic acid, or esters [23–24] besides allylsilane. For example, Angst reported the coupling of styrylsilanes with *N*-acyl-2-chloroglycine esters catalyzed by SnCl_4_ to give the 3-styryl glycine derivatives in 1987 [19]; Wistrand reported the coupling of methyl 1-acyl-5-methoxy-*L*-proline with 1-alkenylcoppers catalyzed by BF_3_·OEt_2_ to give methyl 1-acyl-5-(1-alkenyl)-*L*-proline in 1992 [20]; Menicagli reported the coupling of *N*-acylisoquinolium chloride with di-isobutyl 1-hexenylalanes to give 1,2-dihydro-2-acyl-1-hexenylisoquinolines in 2008 [22]; Schaus reported the coupling of 1-alkenylboronates with 2-ethoxy-*N*-acylquinolines catalyzed by tartaric acid to produce 2-(1-alkenyl)-*N*-acylquinolines in 2011 [23]. We report here a concise synthesis of 3-(1-alkenyl)isoindolin-1-ones and 5-(1-alkenyl)pyrrol-2-ones by the cross-coupling reactions of *N*-acyliminium ions derived from 3-hydroxyisoindol-1-ones or 5-hydroxypyrrol-2-ones with unactivated olefins such as styrene (**2a**) (Scheme 1 and Scheme 2).

Isoindolinones and pyrrolones are the core structures of numerous natural alkaloids [25–27] as well as many drug candidates [28–30]. Isoindolinones demonstrate a remarkably wide range of biological activities, including anti-inflammatory, antihypertensive, antipsychotic and antileukemic and antiviral effects [31–33]. Thus, many methods have been developed to synthesize 2- or 3-functionalized isoindolinones. Among them, only two reports dealt with the synthesis of 3-(1-alkenyl)isoindolin-1-one derivatives. One was, as mentioned above, by the Cp_2_ZrCl_2_ catalyzed coupling of *N*-acyliminium ions with in situ generated dimethyl 1-alkenylalanes [24], another was performed by the palladium-catalyzed coupling of 2-iodobenzoyl chloride with aldimines and subsequent cyclization [34]. The results of our investigation have furnished another route to the synthesis of 3-(1-alkenyl)isoindolin-1-ones and 5-(1-alkenyl)pyrrol-2-ones.

## Results and Discussion

Two kinds of *N*-acyliminium ion precursors, 3-hydroxyisoindol-1-ones (**1a**–**c**) and 5-hydroxypyrrol-2-ones (**5a**,**b**) were easily prepared by the reduction of the parent phthalimide [35] and maleimide [36] derivatives. In order to explore the effects of the experimental conditions on the coupling reactions, the reaction of **1a** with styrene (**2a**) was selected as a representative and carried out at room temperature under different conditions (Table 1). The use of a larger amount of catalyst led to an increase in the yield of the coupling product **3a** (Table 1, entries 1–3). This observation is general for most of the intermolecular coupling reactions of *N*-acyliminium ions with the weakest nucleophiles [14–16]. Of the catalysts examined, BF_3_·OEt_2_ was very efficient for the formation of **3a** compared to other catalysts such as CF_3_SO_3_H, CH_3_CO_2_H, TiCl_4_, SnCl_4_ and InCl_3_. Among various solvents tested, anhydrous dichloromethane (DCM) appeared to be the best choice, providing the desired adduct in the highest yield (>80%). Thus, the reaction employing 2.0 equiv BF_3_·OEt_2_ as catalyst and anhydrous DCM as solvent at room temperature was selected as the model for the general conditions for all of the other reactions.

**Table 1:** Optimization of the intermolecular coupling reaction of **1a** with **2a.**^a^

Entry	Solvent	Catalyst	*t* (h)	*T* (°C)	Yield^b^ (%)

1	CH_2_Cl_2_	1.0 equiv BF_3_·OEt_2_	1.0	25	65
2	CH_2_Cl_2_	1.5 equiv BF_3_·OEt_2_	1.0	25	80
3	CH_2_Cl_2_	2.0 equiv BF_3_·OEt_2_	1.0	25	83
4	CH_2_Cl_2_	2.0 equiv CF_3_SO_3_H	1.0	25	50
5	CH_2_Cl_2_	2.0 equiv CF_3_CO_2_H	1.0	25	37
6	CH_2_Cl_2_	2.0 equiv TiCl_4_	1.0	25	30
7	CH_2_Cl_2_	2.0 equiv SnCl_4_	1.0	25	25
8	CH_2_Cl_2_	2.0 equiv InCl_3_	1.0	25	21
9	CH_3_CN	2.0 equiv BF_3_·OEt_2_	1.0	25	66
10	Et_2_O	2.0 equiv BF_3_·OEt_2_	1.0	25	64

^a^Reactions were carried out on 1.0 mmol scale in 15.0 mL of solvent for 1.0 h with **1a** (0.1 mmol), **2a** (2.0 mmol) and catalyst (2.0 mmol); ^b^isolated yields based on **1a**.

**Scheme 3:**
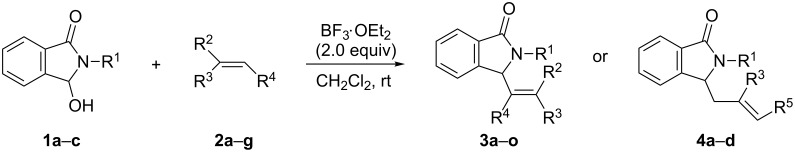
Reactions of 5-hydroxyisoindol-1-ones with olefins in the presence of BF_3_·OEt_2_.

**Figure 1:**
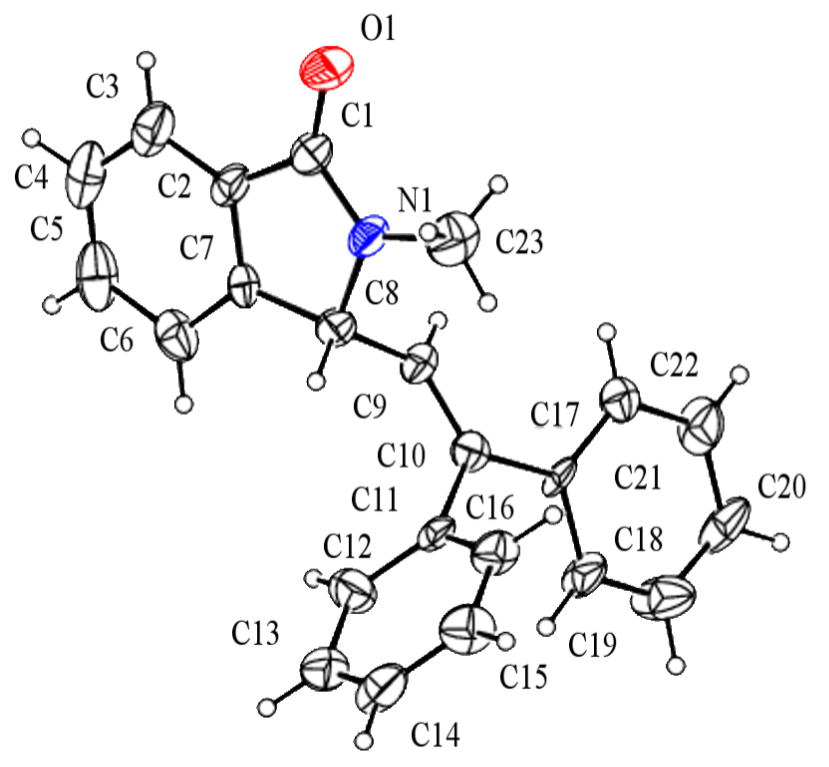
X-Ray structure (ORTEP drawing) of **3h**.

Under the selected conditions, the reactions of substrates **1a**–**c** with different olefins, such as styrene (**2a**), α-methylstyrene (**2b**), 1,1-diphenylethene (**2c**), indene (**2d**), cyclohexene (**2e**), 3,4-dihydropyran (**2f**), 2,3-dihydofuran (**2g**) and 1-hexene (**2h**), were examined (Scheme 3). All reactions proceeded quickly to afford the corresponding coupling products **3a**–**o** or **4a**–**d** in moderate to high yields (Table 2 and Table 3). The products were fully characterized by ^1^H, ^13^C NMR and HRMS, and the structure of **3h** was further confirmed by X-ray crystallography (Figure 1).

**Table 2:** The reactions of 3-hydroxyisoindol-1-one **1a** with olefins **2** in the presence of BF_3_·OEt_2_.^a^

Entry	Reactants						*t* (h)	*T* (°C)	Product		Yield^b^ (%)
					
		R^1^		R^2^	R^3^	R^4^			R^5^		

1	**1a**	PhCH_2_	**2a**	H	Ph	H	0.5	25	—	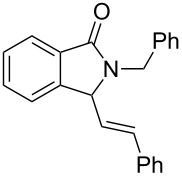 **3a**	83
2	**1a**	PhCH_2_	**2b**	CH_3_	Ph	H	0.5	25	H	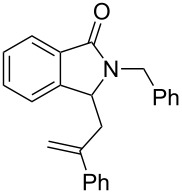 **4a**	90
3	**1a**	PhCH_2_	**2c**	Ph	Ph	H	0.25	25	—	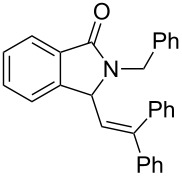 **3b**	93
4	**1a**	PhCH_2_	**2d**	H	–CH_2_C_6_H_4_–		1.0	25	—	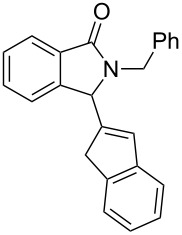 **3c**	78
5	**1a**	PhCH_2_	**2e**	H	–(CH_2_)_4_–		1.0	25	—	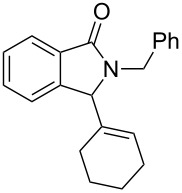 **3d**	77
6	**1a**	PhCH_2_	**2f**	H	–(CH_2_)_3_O–		1.0	25	—	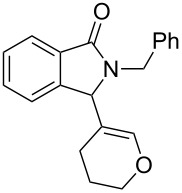 **3e**	59
7	**1a**	PhCH_2_	**2g**	H	–(CH_2_)_2_O–		1.0	25	—	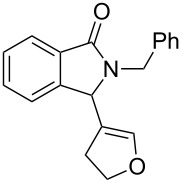 **3f**	54
8	**1a**	PhCH_2_	**2h**	H	H	*n*-Bu	2.0	25	*n*-Pr	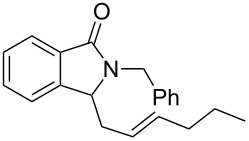 **4b**	47

^a^All reactions were performed under the optimal conditions; ^b^isolated yields based on **1a**.

**Table 3:** The reactions of 3-hydroxyisoindol-1-one (**1b**,**c**) with olefins **2** in the presence of BF_3_·OEt_2_.^a^

Entry	Reactants						*t* (h)	*T* (°C)	Product		Yield^b^ (%)
					
		R^1^		R^2^	R^3^	R^4^			R^5^		

1	**1b**	CH_3_	**2a**	H	Ph	H	0.5	25	—	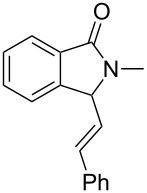 **3g**	70
2	**1b**	CH_3_	**2b**	CH_3_	Ph	H	0.5	25	H	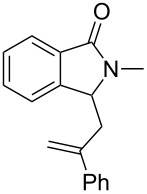 **4c**	93
3	**1b**	CH_3_	**2c**	Ph	Ph	H	0.25	25	—	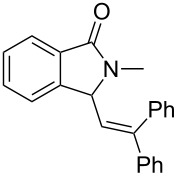 **3h**	94
4	**1b**	CH_3_	**2d**	H	–CH_2_C_6_H_4_–		0.5	25	—	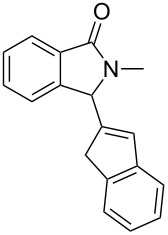 **3i**	65
5	**1b**	CH_3_	**2e**	H	–(CH_2_)_4_–		1.0	25	—	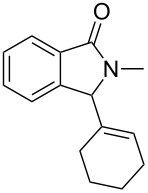 **3j**	53
6	**1b**	CH_3_	**2f**	H	–(CH_2_)_3_O–		1.0	25	—	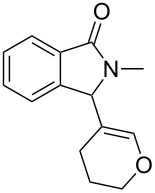 **3k**	48
7	**1b**	CH_3_	**2g**	H	–(CH_2_)_2_O–		1.0	25	—	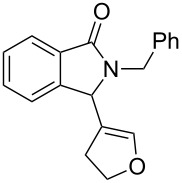 **3l**	45
8	**1c**	H	**2a**	H	Ph	H	1.0	25	*—*	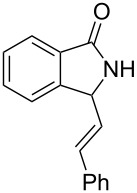 **3m**	58
9	**1c**	H	**2b**	CH_3_	Ph	H	1.0	25	H	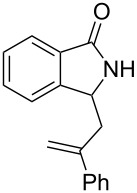 **4d**	66
10	**1c**	H	**2c**	Ph	Ph	H	1.0	25	—	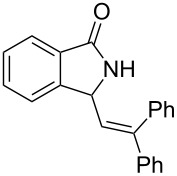 **3n**	73
11	**1c**	H	**2d**	H	–CH_2_C_6_H_4_–		1.0	25	—	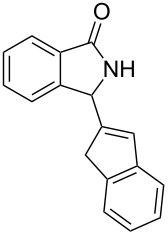 **3o**	50

^a^All reactions were performed under the optimal conditions; ^b^isolated yields based on **1b**,**c**.

It can be seen from Table 2 and Table 3 that substituents such as the benzyl and methyl group at the *N*-atom in **1a**,**b** favored the formation of the coupling products and, thus, higher yields of product were produced from **1a**,**b**. Moreover, both the reaction efficiency and selectivity appeared to be strongly dependent upon variation of the structure of the alkene component. The yields of the coupling adducts are seen to gradually decrease as the nucleophilicity of the alkene diminishes, as is exemplified by the yields recorded for the reactions between **1a** and **1b** and diphenyl ethylene, α-methylstyrene and styrene (case of **1a**: Table 2, entries 1–3 and case of **1b**: Table 3, entries 1–3). The same trend is also observed in the less favorable case of **1c** (Table 3, entries 8–10). Consistent with this reactivity profile, hexene gave only a moderate yield of adduct **4b** when reacted with **1a** (Table 2, entry 8). Likewise, alkenes bearing allylic protons prone to β-elimination, such as α-methylstyrene and hexane, did not afford the “normal” C_sp3_–C_sp2_ vinylic adducts of type **3**, but instead the C_sp3_–C_sp3_ coupling products **4** were isolated (Table 2, entries 2 and 8 and Table 3, entries 2 and 9) much like the ene-type adducts of oxonium ion with olefins [37–38]. This means that these alkenes may be envisioned as surrogates of their corresponding, more expensive and less atom-economical, allylsilane derivatives, which are typically used in *N*-acyliminium ion chemistry to produce amide compounds substituted with an α-allyl group. The reactions of cyclic alkenes (**2d**–**g**) with **1a**,**b** all gave the normal C_sp3_–C_sp2_ coupling products in moderate yields.

**Scheme 4:**
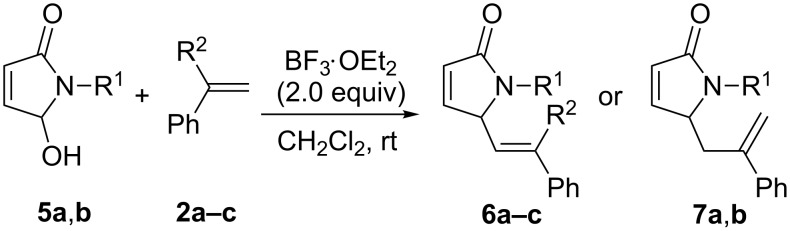
Reactions of 5-hydroxypyrrol-1-ones with olefins in the presence of BF_3_·OEt_2_.

The coupling reactions were examined under the same conditions with alternate substrates (**5a**,**b**), and olefins (**2a**–**c**) (Scheme 4). All these reactions gave the cross-coupling products (Table 4). As compared with **1a**–**c**, the rates of the coupling reactions of **5a**,**b** with **2a**–**c** were somewhat slower and the yields of the corresponding products were also decreased, probably as a result of both the limited nucleophilicity parameter of the alkenes [39] and the lower stability of the transient *N*-acyliminium intermediate derived from **5a**,**b**. Similarly to the reactions of **1a**–**c** with α-methylstyrene (**2b**), the reactions of **5a**,**b** with **2b** also gave the C_sp3_–C_sp3_ coupling products **7a**,**b** instead of the C_sp3_–C_sp2_ coupling products.

**Table 4:** The reactions of 5-hydroxypyrrol-2-ones **5** with olefins **2** in the presence of BF_3_·OEt_2_.^a^

Entry	Reactants					*t* (h)	*T* (°C)	Product	Yield^b^ (%)
					
		R^1^		R^2^	R^3^				

1	**5a**	PhCH_2_	**2a**	H	Ph	2.0	25	**6a**	55
2	**5a**	PhCH_2_	**2b**	CH_3_	Ph	2.0	25	**7a**	82
3	**5a**	PhCH_2_	**2c**	Ph	Ph	2.0	25	**6b**	72
4	**5b**	CH_3_	**2a**	CH_3_	Ph	2.0	25	**7b**	72
5	**5b**	CH_3_	**2b**	Ph	Ph	2.0	25	**6c**	65

^a^All reactions were performed under the optimal conditions; ^b^isolated yields based on **5a**,**b**.

## Conclusion

In summary, we have developed a concise route for the synthesis of 3-(1-alkenyl)isoindolin-1-ones and 5-(1-alkenyl)pyrrol-2-ones by the coupling reactions of *N*-acyliminium ions derived from 3-hydroxyisoindol-1-ones or 5-hydroxypyrrol-2-ones with unactivated olefins in the presence of BF_3_·OEt_2_ at room temperature. For most of the olefins, the reactions afforded the C_sp3_–C_sp2_ cross-coupling products, but for α-methylstyrene and 1-hexene, the C_sp3_–C_sp3_ cross-coupling products were produced.

## Experimental

### General information

All reagents were purchased from commercial suppliers and used without further purification. All solvents were dried and redistilled before use. Flash chromatography was carried out with silica gel (200–300 mesh). Analytical TLC was performed with silica gel GF_254_ plates, and the products were visualized by UV detection. Melting points were determined on a Yanagimoto melting point apparatus and are uncorrected. ^1^H and ^13^C NMR spectra were recorded on a Bruker AM-400 NMR or a Bruker DRX-300 NMR spectrometer in CDCl_3_ with TMS as an internal standard. EIMS were recorded with a HP 5988 A mass spectrometer. HRMS (ESI) were measured on a Bruker Dattonics APEX 47e mass spectrometer.

### General procedure for the coupling reactions

To a solution of **1a** (1.0 mmol) and olefin **2a** (2.0 mmol) in 15 mL of anhydrous methylene dichloride, BF_3_·OEt_2_ (2.0 mmol) was added at 25 °C in one portion under stirring. After continued stirring at 25 °C until **1a** disappeared (monitored by TLC), the reaction was quenched with water. The mixture was separated and the aqueous phase was extracted with methylene dichloride (10 mL). The combined organic layers were washed with water (20 mL), dried with anhydrous Na_2_SO_4_ and concentrated in vacuo. The residue was separated by silica-gel column chromatography, eluted by hexane/acetone (10:1 v/v), to give the corresponding product **3a**.

**(*E*)-2-Benzyl-3-(2-phenylethenyl)isoindolin-1-one (3a):** Colorless syrup; ^1^H NMR (400 MHz, CDCl_3_) δ 4.22 (d, *J* = 14.8 Hz, 1H), 4.90 (d, *J* = 9.2 Hz, 1H), 5.33 (d, *J* = 14.8 Hz, 1H), 5.82 (dd, *J* = 9.2 Hz, 15.6 Hz, 1H), 6.77 (d, *J* = 15.6 Hz, 1H), 7.28–7.37 (m, 11H), 7.50–7.55 (m, 2H), 7.92 (dd, *J* = 1.6 Hz, 6.4 Hz, 1H) ppm; ^13^C NMR (100 MHz, CDCl_3_) δ 44.1, 62.7, 123.2, 123.8, 125.6, 126.7 (2C), 127.5, 128.4 (2C), 128.5 (2C), 128.6 (2C), 128.7, 128.7, 131.7, 131.8, 135.7, 135.9, 137.4, 144.5, 168.0 (CO) ppm; EIMS *m*/*z* (% relative intensity): 325 (56), 310 (29), 234 (89), 220 (31), 149 (46), 91 (45), 57 (53), 44 (100); HRMS–ESI (*m*/*z*): [M + H]^+^ calculated for C_23_H_20_NO, 326.1540; found, 326.1536.

**2-Benzyl-3-(2-phenyl-2-propenyl)isoindolin-1-one (4a):** Colorless solid, mp 69–72 °C; ^1^H NMR (400 MHz, CDCl_3_) δ 2.54 (dd, *J* = 9.2 Hz, 14.0 Hz, 1H), 3.40 (dd, *J* = 4.0 Hz, 14.0 Hz, 1H), 4.24 (d, *J* = 15.6 Hz, 1H), 4.39 (dd, *J* = 4.0 Hz, 9.2 Hz, 1H), 5.00 (s, 1H), 5.38 (s, 1H), 5.40 (d, *J* = 15.6 Hz, 1H), 7.20–7.31 (m, 11H), 7.41 (t, *J* = 4.0 Hz, 2H), 7.86 (t, *J* = 4.0 Hz, 1H) ppm; ^13^C NMR (100 MHz, CDCl_3_) δ 38.1, 44.1, 56.9, 116.9, 123.2, 123.7, 126.1 (2C), 127.6, 127.8, 128.1, 128.1 (2C), 128.5 (2C), 128.8 (2C), 130.9, 131.8, 137.0, 139.8, 143.6, 145.2, 168.4 (CO) ppm; MS *m*/*z* (% relative intensity): 339 (1), 253 (4), 237 (6), 222 (100), 197 (5), 149 (13), 91 (71); HRMS–ESI (*m*/*z*): [M + H]^+^ calcd for C_24_H_22_NO, 340.1696; found, 340.1699.

**2-Benzyl-3-cyclohexenylisoindolin-1-one (3d):** Colorless solid, mp 109–112 °C; ^1^H NMR (400 MHz, CDCl_3_) δ 1.15–1.19 (m, 1H), 1.38–1.43 (m, 3H), 1.50–1.59 (m, 2H), 2.13 (t, *J* = 2.4 Hz, 2H), 4.06 (d, *J* = 14.8 Hz, 1H), 4.71 (s, 1H), 5.19 (d, *J* = 14.8 Hz, 1H), 5.93 (s, 1H), 7.26–7.30 (m, 5H), 7.41–7.50 (m, 3H), 7.87 (d, *J* = 7.2 Hz, 1H) ppm; ^13^C NMR (100 MHz, CDCl_3_) δ 21.8, 22.0, 22.2, 25.4, 43.9, 66.7, 122.4, 123.4, 127.3, 128.1, 128.4 (4C), 130.2, 131.4, 132.3, 133.4, 137.4, 144.4, 168.3 (CO) ppm; MS *m*/*z* (% relative intensity): 303 (64), 222 (27), 199 (70), 183 (6), 170 (12), 157 (15), 129 (27), 91 (100), 40 (37); HRMS–ESI (*m*/*z*): [M + H]^+^ calcd for C_21_H_22_NO, 304.1696; found, 304.1691.

**(*E*)-2-Benzyl-3-(hex-2-enyl)isoindolin-1-one (4b):** Colorless syrup; ^1^H NMR (400 MHz, CDCl_3_) δ 0.74 (t, *J* = 7.2 Hz, 3H), 1.25–1.87 (m, 2H), 1.79–1.86 (m, 2H), 2.55–2.70 (m, 2H), 4.17 (d, *J* = 15.2 Hz, 1H), 4.39 (dd, *J* = 4.0 Hz, 5.6 Hz, 1H), 4.91–4.98 (m, 1H), 5.36–5.42 (m, 1H), 5.42 (d, *J* = 15.2 Hz, 1H), 7.28–7.32 (m, 5H), 7.37 (d, *J* = 7.2 Hz, 1H), 7.43–7.53 (m, 2H), 7.88 (d, *J* = 7.6 Hz, 1H) ppm; ^13^C NMR (100 MHz, CDCl_3_) δ 13.4, 22.3, 34.1, 34.5, 43.9, 58.4, 122.4 (2C), 123.7, 127.5, 128.0, 128.1 (2C), 128.7 (2C), 131.2, 132.4, 135.4, 137.2, 145.1, 168.5 (CO) ppm; MS *m*/*z* (% relative intensity): 305 (4), 223 (18), 222 (100), 186 (6), 172 (6), 132 (8), 104 (5), 91 (89); HRMS–ESI (*m*/*z*): [M + H]^+^ calcd for C_21_H_24_NO, 306.1853; found, 306.1851.

**3-(2,2-Diphenylethenyl)-2-methylisoindolin-1-one (3h):** Colorless solid, mp 146–148 °C; ^1^H NMR (400 MHz, CDCl_3_) δ 3.08 (s, 3H), 5.01 (d, *J* = 10.0 Hz, 1H), 5.71 (d, *J* = 10.0 Hz, 1H), 7.25–7.27 (m, 5H), 7.38–7.46 (m, 5H), 7.47–7.52 (m, 3H), 7.83 (d, *J* = 7.6 Hz, 1H) ppm; ^13^C NMR (100 MHz, CDCl_3_) δ 27.5, 61.3, 122.8, 123.4, 124.3, 127.2 (2C), 127.9, 128.1, 128.3 (3C), 128.8 (2C), 129.5 (2C), 131.3, 132.3, 138.5, 140.4, 144.5, 148.1, 168.0 (CO) ppm; MS *m*/*z* (% relative intensity): 325 (28), 310 (15), 294 (9), 265 (5), 248 (11), 220 (18), 188 (10), 178 (11), 165 (13), 149 (37), 91 (30), 57 (63), 43 (100); HRMS–ESI (*m*/*z*)*:* [M + H]^+^ calcd for C_23_H_20_NO, 326.1540; found, 326.1545.

**Crystal data for 3h (recrystallized from ethanol):** C_23_H_19_NO, *M*_r_ = 325.39. Monoclinic, *a* = 17.373(11) Å, *b* = 17.241(11) Å, *c* = 24.421(16) Å, β = 91.219(9), *V* = 7313(8) Å^3^, colorless plates, ρ = 1.182 g cm^−3^, *T* = 296(2) K, space group *P*2(1)/*c*, *Z* = 4, μ (Mo Kα) = 0.084 mm^−1^, 2θ_max_ = 51°, 9126 reflections measured, 3995 unique (*R*_int_ = 0.0696), which were used in all calculations. The final w*R(F^2^)* was 0.1427 (for all data), *R*_1_ = 0.0764. CCDC file No. 835330.

**3-(3,4-Dihydro-2*H*-pyran-5-yl)-2-methylisoindolin-1-one (3k):** Colorless solid, mp 94–97 °C; ^1^H NMR (400 MHz, CDCl_3_) δ 1.20–1.27 (m, 1H), 1.40–1.47 (m, 1H), 1.71–1.79 (m, 2H), 3.00 (s, 3H), 3.92–4.04 (m, 2H), 4.65 (s, 1H), 6.79 (s, 1H), 7.36 (d, *J* = 7.2 Hz, 1H), 7.44 (t, *J* = 7.2 Hz, 1H), 7.53 (t, *J* = 7.2 Hz, 1H), 7.81 (d, *J* = 7.2 Hz, 1H) ppm; ^13^C NMR (100 MHz, CDCl_3_) δ 17.0, 21.5, 26.6, 65.2, 66.0, 108.2, 122.2, 123.0, 128.1, 131.3, 132.7, 144.2, 144.6, 168.2 (CO) ppm; MS *m*/*z* (% relative intensity): 229 (100), 200 (47), 186 (35), 172 (54), 146 (51), 128 (20), 115 (17), 91 (24); HRMS–ESI (*m*/*z*): [M + H]^+^ calcd for C_14_H_16_NO_2_, 230.1176; found, 230.1175.

**(*E*)-1-Benzyl-5-(2-phenylethenyl)-1*H*-pyrrol-2(5*H*)-one (6a):** Colorless syrup; ^1^H NMR (400 MHz, CDCl_3_) δ 4.08 (d, *J* = 14.8 Hz, 1H), 4.54 (d, *J* = 9.2 Hz, 1H), 5.12 (d, *J* = 14.8 Hz, 1H), 5.69 (dd, *J* = 9.2 Hz, 15.6 Hz, 1H), 6.26 (dd, *J* = 1.6 Hz, 5.6 Hz, 1H), 6.59 (d, *J* = 15.6 Hz, 1H), 6.96 (dd, *J* = 1.6 Hz, 6.0 Hz, 1H), 7.23–7.35 (m, 8H), 7.40 (dd, *J* = 1.6 Hz, 8.0 Hz, 2H) ppm; ^13^C NMR (100 MHz, CDCl_3_) δ 42.4, 64.8, 126.1, 126.6, 127.4, 128.0, 128.2, 128.6 (2C), 128.7 (2C), 128.7 (2C), 128.9 (2C), 135.7, 137.6, 146.6, 170.9 (CO) ppm; MS *m*/*z* (% relative intensity): 275 (22), 190 (11), 189 (100), 184 (30), 161 (29), 160 (39), 132 (37), 119 (22), 104 (48), 91 (21); HRMS–ESI (*m*/*z*): [M + H]^+^ calcd for C_19_H_18_NO, 276.1383; found, 276.1385.

## Supporting Information

File 1 Characterization data of the title compounds, ^1^H NMR and ^13^C NMR spectra.

File 2 X-ray data for compound **3h**.
