# Supplementary material for: A concise synthesis of 3-(1-alkenyl)isoindolin-1-ones and 5-(1-alkenyl)pyrrol-2-ones by the intermolecular coupling reactions of N-acyliminium ions with unactivated olefins
Source: Beilstein J Org Chem. 2012 Feb 6;8:192–200. doi: 10.3762/bjoc.8.21 (PMC3302081; doi:10.3762/bjoc.8.21)

Supporting Information

for

A concise synthesis of 3-(1-alkenyl)isoindolin-1-ones and 5-(1-alkenyl)pyrrol-2-ones by the intermolecular coupling reactions of *N*-acyliminium ions with unactivated olefins

Nianhong Lu, Lihong Wang, Zhanshan Li, Wei Zhang*

Address: State Key Laboratory of Applied Organic Chemistry, Lanzhou University, Lanzhou 730000, China; Fax: +86 (931) 8625657

Email: Wei Zhang - zhangwei6275@lzu.edu.cn

*Corresponding author

Characterization data of title compounds, ^{1}H NMR and ^{13}C NMR spectra

Content

General remarks S2

Experimental procedures S2

References S3

Analytical data S4

^{1}H NMR and ^{13}C NMR spectra S10

General remarks

All reagents were purchased from commercial suppliers and used without further purification. Flash chromatography was carried out with silica gel (200–300 mesh). Analytical TLC was performed with silica gel GF254 plates, and the products were visualized by UV detection. ^{1}H NMR and ^{13}C NMR (400 MHz and 100 MHz, respectively) spectra were recorded in $CDCl_3$. Chemical shifts (δ) are reported in ppm with TMS as internal standard, and spin–spin coupling constants (*J*) are given in Hz. EIMS were recorded with a HP 5988 A mass spectrometer. HRMS (ESI) were measured on a Bruker Dattonics APEX 47e mass spectrometer.

Experimental procedures

1. General procedure for the preparation of *N*-benzylphthalimide, *N*-methylphthalimide and *N*-benzyl-1*H*-pyrrol-2,5-dione [1]

To a solution of the corresponding anhydride (10 mmol) in glacial acetic acid (4 mL) was added slowly the primary amine (12 mmol). The mixture was heated under reflux for a given time until the corresponding anhydride disappeared (monitored by TLC). The reaction was quenched with ice water. The mixture was filtered and the colorless solid was washed with distilled water. The solid was dried under vacuum (70 mbar) at 80 °C and then recrystallized from ethanol to give *N*-benzylphthalimide (*N*-methylphthalimide and *N*-benzyl-1*H*-pyrrole-2,5-dione) as colorless crystals (80–98%).

2. General procedure for the preparation of *N*-substituted 3-hydroxy-isoindolin-1-one (1a–c) [2]

1.0 mmol of *N*-substituted phthalimides were stirred at 0 °C in anhydrous MeOH (5 mL) and THF (10 mL) for 10 min. $NaBH_4$ (1.0 mmol) was added slowly over 5–10 min, the mixture was stirred at 0 °C until the *N*-substituted phthalimides disappeared (monitored by TLC). The reaction was quenched with water, and the mixture was extracted with ethyl acetate. The combined organic layers were dried with anhydrous Na_2SO_4, and concentrated in vacuo. The residue was separated by silica-gel column chromatography to obtain the corresponding products (70–85%).

3. General procedure for the preparation of *N*-substituted 5-hydroxy-1*H*-pyrrol-2(5*H*)-one (5a,b) [2]

1.0 mmol of *N*-substituted-1*H*-pyrrol-2,5-dione and 1.0 mmol of $CeCl_3 \cdot 7H_2O$ were stirred at 0 °C in anhydrous MeOH (5 mL) and THF (10 mL) for 10 min. $NaBH_4$ (1.0 mmol) was added slowly over 5–10 min, the mixture was stirred at 0 °C until the 1-substitued-1*H*-pyrrole-2,5-dione disappeared (monitored by TLC). The reaction was quenched with water, and the mixture was extracted with ethyl acetate. The combined organic layers were dried with anhydrous Na_2SO_4, and concentrated in vacuo. The residue was separated by silica-gel column chromatography to obtain the corresponding products **5a** and **5b** (70–85% and 40–50%, respectively).

4. General procedure for the coupling reactions

To a solution of **1a–c** or **5a**,**b** (1.0 mmol) and olefins (2.0 mmol) in 15 mL of processed methylene dichloride was added $BF_3 \cdot OEt_2$ (2.0 mmol) in one portion under stirring at 25 ℃. Stirring was continued for a given time until the **1a–c** or **5a**,**b** disappeared (monitored by TLC). The reaction was quenched with water, and the mixture was separated. The aqueous phase was extracted with methylene dichloride (10 × 2 mL). The combined organic layer was dried with anhydrous Na_2SO_4, and concentrated in vacuo. The residue was separated by silica-gel column chromatography to obtain the corresponding products **3a–o**, **4a–d**, **6a–c** and **7a,b** as summarized in Table 2–Table 4 in the main article.

References

(1) Godt, A.; Sajid, M.; Jeschke, G. *Chem. Eur J.* **2009**, 15(47), 12961;

(2) (a)Wei Zhang; Airong Zheng; Zhenang Liu. *Tetrahedron Lett.* **2005,** 46, 5693;

(b) Wei Zhang; Liming Huang; Junpu Wang. *Synthesis*. **2006,** 12, 2056;

(c) Wei Zhang; Yuehua Zhou; Lingfeng Qiang. *Synlett*. **2009,** 5, 846;

(d) Wei Zhang; Lingfeng Qiang; Yuehua Zhou. *Chin. Chem. Lett.* **2009,** 20, 807.

Analytical data of products

(*E*)-2-Benzyl-3-(2-phenylethenyl)isoindolin-1-one (**3a**)
Colorless syrup. ^{1}H NMR (400 MHz, $CDCl_3$) δ ppm 4.22 (d, *J* = 14.8 Hz, 1H), 4.90 (d, *J* = 9,2 Hz, 1H), 5.33 (d, *J* = 14.8 Hz, 1H), 5.82 (dd, *J* = 9.2 Hz, 15.6 Hz, 1H), 6.77 (d, *J* = 15.6 Hz, 1H), 7.28–7.37 (m, 11H), 7.50–7.55 (m, 2H), 7.92 (dd, *J* = 1.6 Hz, 6.4 Hz, 1H); ^{13}C NMR (100 MHz, $CDCl_3$): δ ppm 44.1, 62.7, 123.2, 123.8, 125.6, 126.7 (2C), 127.5, 128.4 (2C), 128.5 (2C), 128.6 (2C), 128.7, 128.7, 131.7, 131.8, 135.7, 135.9, 137.4, 144.5, 168.0 (CO). EIMS *m*/*z* (relative intensity, %): 325 (56), 310 (29), 234 (89), 220 (31), 149 (46), 91 (45), 57 (53), 44 (100); HRMS–ESI (*m*/*z*) calcd for $C_{23}H_{19}NO+H^+$, 326.1540; found, 326.1536.

2-Benzyl-3-(2-phenyl-2-propenyl)isoindolin-1-one (**4a**)
Colorless solid; mp 69–72 °C; ^{1}H NMR (400 MHz, $CDCl_3$) δ ppm 2.54 (dd, *J* = 9.2 Hz, 14.0 Hz, 1H), 3.40 (dd, *J* = 4.0 Hz, 14.0 Hz, 1H), 4.24 (d, *J* = 15.6 Hz, 1H), 4.39 (dd, *J* = 4.0 Hz, 9.2 Hz, 1H), 5.00 (s, 1H), 5.38 (s, 1H), 5.40 (d, *J* = 15.6 Hz, 1H), 7.20–7.31 (m, 11H), 7.41 (t, *J* = 4.0 Hz, 2H), 7.86 (t, *J* = 4.0 Hz, 1H); ^{13}C NMR (100 MHz, $CDCl_3$) δ ppm 38.1, 44.1, 56.9, 116.9, 123.2, 123.7, 126.1 (2C), 127.6, 127.8, 128.1, 128.1 (2C), 128.5 (2C), 128.8 (2C), 130.9, 131.8, 137.0, 139.8, 143.6, 145.2, 168.4 (CO); EIMS *m*/*z* (relative intensity, %): 339 (1), 253 (4), 237 (6), 222 (100), 197 (5), 149 (13), 91 (71); HRMS–ESI (*m*/*z*) calcd for $C_{24}H_{21}NO+H^+$, 340.1696; found, 340.1699.

2-Benzyl-3-(2,2-diphenylethenyl)isoindolin-1-one (**3b**)
Colorless solid; mp 134–136 °C; ^{1}H NMR (400 MHz, $CDCl_3$) δ ppm 4.39 (d, *J* = 15.2 Hz, 1H), 5.10 (d, *J* = 15.2 Hz, 1H), 5.12 (d, *J* = 10.0 Hz, 1H), 5.64 (d, *J* = 10.0 Hz, 1H), 7.07–7.30 (m, 15H), 7.41–7.53 (m, 3H), 7.89 (d, *J* = 7.6 Hz, 1H); ^{13}C NMR (100 MHz, $CDCl_3$) δ ppm 44.4, 59.4, 123.1, 123.8, 124.5, 127.2, 127.3 (2C), 127.7, 127.8 (2C), 128.0, 128.2 (2C), 128.4, 128.5 (4C), 129.5 (2C), 131.6, 132.0, 137.5, 138.2, 140.8, 144.8, 147.8, 168.2 (CO); EIMS *m*/*z* (relative intensity, %): 401 (2), 223 (6), 205 (7), 178 (8), 149 (91), 97 (36), 57 (47), 43 (68) 40 (100); HRMS–ESI (*m*/*z*) calcd for $C_{29}H_{23}NO+H^+$, 402.1853; found, 402.1851.

2-Benzyl-3-(1*H*-inden-2-yl)isoindolin-1-one (**3c**)
Pale yellow syrup; ^{1}H NMR (400 MHz, $CDCl_3$) δ ppm 2.79 (d, *J* = 22.8 Hz, 1H), 3.06 (d, *J* = 22.8 Hz, 1H), 3.95 (d, *J* = 14.8 Hz, 1H), 5.34 (s, 1H), 5.38 (d, *J* = 14.8 Hz, 1H), 7.03 (s, 1H), 7.16–7.20 (m, 1H), 7.25–7.32 (m, 8H), 7.42 (d, *J* = 7.6 Hz, 1H), 7.47–7.49 (m, 2H), 7.93–7.95 (m, 1H); ^{13}C NMR (100 MHz, $CDCl_3$) δ ppm 36.1, 44.1, 60.1, 121.2, 122.9, 123.9, 124.0, 125.4, 126.6, 127.6, 128.4 (2C), 128.5 (2C), 128.7, 131.7, 131.8, 133.5, 137.1, 143.4, 143.6, 144.0, 144.9, 168.1 (CO); EIMS *m*/*z* (relative intensity, %): 337 (17), 258 (12), 246 (23), 228 (46), 178 (23), 155 (65), 149 (84), 139 (63), 129 (65), 111 (51), 97 (32), 91 (39), 86 (50), 71 (63), 57 (100), 43 (87); HRMS–ESI (*m*/*z*) calcd for $C_{24}H_{19}NO+H^+$, 338.1540; found, 338.1537.

2-Benzyl-3-cyclohexenylisoindolin-1-one (**3d**)

Colorless solid; mp 109–112 °C; ^{1}H NMR (400 MHz, $CDCl_3$) δ ppm 1.15–1.19 (m, 1H), 1.38–1.43 (m, 3H), 1.50–1.59 (m, 2H), 2.13 (t, *J* = 2.4 Hz, 2H), 4.06 (d, *J* = 14.8 Hz, 1H), 4.71 (s, 1H), 5.19 (d, *J* = 14.8 Hz, 1H), 5.93 (s, 1H), 7.26–7.30 (m, 5H), 7.41–7.50 (m, 3H), 7.87 (d, *J* = 7.2 Hz, 1H); ^{13}C NMR (100 MHz, $CDCl_3$) δ ppm 21.8, 22.0, 22.2, 25.4, 43.9, 66.7, 122.4, 123.4, 127.3, 128.1, 128.4 (4C), 130.2, 131.4, 132.3, 133.4, 137.4, 144.4, 168.3 (CO); EIMS *m*/*z* (relative intensity, %): 303 (64), 222 (27), 199 (70), 183 (6), 170 (12), 157 (15), 129 (27), 91 (100), 40 (37); HRMS–ESI (*m*/*z*) calcd for $C_{21}H_{21}NO+H^+$, 304.1696; found, 304.1691.

2-Benzyl-3-(3,4-dihydro-2*H*-pyran-5-yl)isoindolin-1-one (**3e**)

Colorless solid; mp 96–98 °C; ^{1}H NMR (400 MHz, $CDCl_3$) δ ppm 1.20–125 (m, 1H), 1.35–1.43 (m, 1H), 1.67 (t, *J* = 5.6 Hz, 2H), 3.88–4.00 (m, 2H), 4.13 (d, *J* = 14.4 Hz, 1H), 4.62 (s, 1H), 5.18 (d, *J* = 14.4 Hz, 1H), 6.62 (s, 1H), 7.26–7.32 (m, 5H), 7.44–7.54 (m, 3H), 7.88 (d, *J* = 7.2 Hz, 1H); ^{13}C NMR (100 MHz, $CDCl_3$) δ ppm 17.3, 21.6, 43.8, 63.0, 66.2, 108.1, 122.5, 123.6, 127.4, 128.3, 128.5 (2C), 128.6 (2C), 131.6, 132.6, 137.6, 144.5, 145.1, 168.3 (CO); EIMS *m*/*z* (relative intensity, %): 305 (60), 237 (15), 221 (28), 214 (35), 200 (93), 186 (24), 172 (40), 149 (79), 129 (27), 91 (100), 71 (40), 57 (53); HRMS–ESI (*m*/*z*) calcd for $C_{20}H_{19}NO_2+H^+$, 306.1489; found, 306.1492.

2-Benzyl-3-(4,5-dihydrofuran-3-yl)isoindolin-1-one (**3f**)

Pale yellow syrup; ^{1}H NMR (400 MHz, $CDCl_3$) δ ppm 1.88–1.97 (m, 1H), 2.01–2.10 (m, 1H), 4.17 (d, *J* = 14.8 Hz, 1H), 4.26–4.33 (m, 2H), 5.08 (s, 1H), 5.22 (d, *J* = 14.8 Hz, 1H), 6.58 (s, 1H), 7.30–7.32 (m, 6H), 7.48–7.53 (m, 2H), 7.90 (d, *J* = 7.2 Hz, 1H); ^{13}C NMR (100 MHz, $CDCl_3$) δ ppm 26.9, 43.9, 56.8, 70.9, 110.1, 122.5, 123.8, 127.5, 128.5 (2C), 128.7 (2C), 131.7, 132.3, 136.9, 137.4, 144.0, 146.8, 168.1 (CO); EIMS *m*/*z* (relative intensity, %): 291 (56), 222 (15), 208 (36), 200 (47), 198 (88), 174 (37), 160 (29), 151 (67), 91 (100), 57 (47); HRMS–ESI (*m*/*z*) calcd for $C_{19}H_{17}NO_2+H^+$, 292.1332; found, 292.1335.

(*E*)-2-Benzyl-3-(hex-2-enyl)isoindolin-1-one (4b)

Colorless syrup; ^{1}H NMR (400 MHz, $CDCl_3$) δ ppm 0.74 (t, *J* = 7.2 Hz, 3H), 1.87–1.25 (m, 2H), 1.79–1.86 (m, 2H), 2.55–2.70 (m, 2H), 4.17 (d, *J* = 15.2 Hz, 1H), 4.39 (dd, *J* = 4.0 Hz, 5.6 Hz, 1H), 4.91–4.98 (m, 1H), 5.42 (d, *J* = 15.2 Hz, 1H), 5.36–5.42 (m, 1H), 7.28–7.32 (m, 5H), 7.37 (d, *J* = 7.2 Hz, 1H), 7.43–7.53 (m, 2H), 7.88 (d, *J* = 7.6 Hz, 1H); ^{13}C NMR (100 MHz, $CDCl_3$) δ ppm 13.4, 22.3, 34.1, 34.5, 43.9, 58.4, 122.4 (2C), 123.7, 127.5, 128.0, 128.1 (2C), 128.7 (2C), 131.2, 132.4, 135.4, 137.2, 145.1, 168.5 (CO); EIMS *m*/*z* (relative intensity, %): 305 (4), 223 (18), 222 (100), 186 (6), 172 (6), 132 (8), 104 (5), 91 (89); HRMS–ESI (*m*/*z*) calcd for $C_{21}H_{23}NO+H^+$, 306.1853; found, 306.1851.

(*E*)-2-Methyl-3-(2-phenylethenyl)isoindolin-1-one (**3g**)

Colorless syrup; ^{1}H NMR (400 MHz, $CDCl_3$) δ ppm 3.12 (s, 3H), 4.94 (d, *J* = 8.8 Hz, 1H), 5.86 (dd, *J* = 8.8 Hz, 15.6 Hz, 1H), 6.91 (d, *J* = 15.6 Hz, 1H), 7.30–7.43 (m, 6H), 7.48 (t, *J* = 7.2 Hz, 1H), 7.53 (t, *J* = 7.2 Hz, 1H), 7.86 (d, *J* = 7.2 Hz, 1H); ^{13}C NMR (100 MHz, $CDCl_3$) δ ppm 27.5, 65.4, 123.0, 123.5, 125.6, 126.7 (2C), 128.5, 128.5, 128.7 (2C), 131.5, 132.1,

135.7, 135.7, 144.3, 168.2 (CO); EIMS *m*/*z* (relative intensity, %): 249 (100), 248 (23), 234 (12), 220 (19), 172 (14), 158 (65) 146 (60), 117 (14), 91 (35), 77 (11), 40 (35); HRMS–ESI (*m*/*z*) calcd for $C_{17}H_{15}NO+H^+$, 250.1227; found, 250.1229.

2-Methyl-3-(2-phenyl-2-propenyl)isoindolin-1-one (**4c**)

Pale yellow syrup; ^{1}H NMR (400 MHz, $CDCl_3$) δ 2.76 ppm (dd, *J* = 7.6 Hz, 14.4 Hz, 1H), 3.08 (s, 3H), 3.25 (dd, *J* = 4.4 Hz, 14.4 Hz, 1H), 4.42 (dd, *J* = 4.4 Hz, 7.6 Hz, 1H), 5.08 (d, *J* = 0.8 Hz, 1H), 5.37 (d, *J* = 0.8 Hz, 1H), 7.28–7.41 (m, 8H), 7.78 (dd, *J* = 1.6 Hz, 6.4 Hz, 1H); ^{13}C NMR (100 MHz, $CDCl_3$) δ ppm 27.8, 38.7, 60.0, 116.8, 122.7, 123.2, 126.1 (2C), 127.8, 127.9, 128.6 (2C), 130.7, 132.0, 140.3, 143.9, 145.0, 168.2 (CO); EIMS *m*/*z* (relative intensity, %): 263 (2), 147 (10), 146 (100), 91 (13), 40 (3); HRMS–ESI (*m*/*z*) calcd for $C_{18}H_{17}NO+H^+$, 264.1383; found, 264.1380.

3-(2,2-Diphenylethenyl)-2-methylisoindolin-1-one (**3h**)

Colorless solid, mp 146–148 °C; ^{1}H NMR (400 MHz, $CDCl_3$) δ ppm 3.08 (s, 3H), 5.01 (d, *J* = 10.0 Hz, 1H), 5.71 (d, *J* = 10.0 Hz, 1H), 7.25–7.27 (m, 5H), 7.38–7.46 (m, 5H), 7.47–7.52 (m, 3H), 7.83 (d, *J* = 7.6 Hz, 1H); ^{13}C NMR (100 MHz, $CDCl_3$) δ ppm 27.5, 61.3, 122.8, 123.4, 124.3, 127.2 (2C), 127.9, 128.1, 128.3 (3C), 128.8 (2C), 129.5 (2C), 131.3, 132.3, 138.5, 140.4, 144.5, 148.1, 168.0 (CO); EIMS *m*/*z* (relative intensity, %): 325 (28), 310 (15), 294 (9), 265 (5), 248 (11), 220 (18), 188 (10), 178 (11), 165 (13), 149 (37), 91 (30), 57 (63), 43 (100); HRMS–ESI (*m*/*z*) calcd for $C_{23}H_{19}NO+H^+$, 326.1540; found, 326.1545.

Crystal data for compound **3h** (recrystallized from ethanol): $C_{23}H_{19}NO$, M_r = 325.39. Monoclinic, a = 17.373(11) Å, b = 17.241(11) Å, c = 24.421(16) Å, β = 91.219(9), V = 7313(8) Å^3, colorless plates, ρ = 1.182 g cm^{-3}, T = 296(2) K, space group $P2(1)/c$, Z = 4, μ (Mo Kα) = 0.084 mm^{-1}, $2\theta_{max}$ = 51°, 9126 reflections measured, 3995 unique (R_{int} = 0.0696), which were used in all calculations. The final w$R(F^2)$ was 0.1427 (for all data), R_1 = 0.0764. CCDC file No. 835330.

3-(2,3-Dihydro-1*H*-inden-2-yl)-2-methylisoindolin-1-one (**3i**)

Pale yellow solid; mp 132–134 °C; ^{1}H NMR (400 MHz, $CDCl_3$) δ ppm 2.81 (d, *J* = 22.8 Hz, 1H), 3.05 (s, 3H), 3.07 (d, *J* = 22.8 Hz, 1H), 5.40 (s, 1H), 7.14 (s, 1H), 7.18 (dt, *J* = 0.8 Hz, 7.2 Hz, 1H), 7.26–7.32 (m, 3H), 7.41 (d, *J* = 7.6 Hz, 1H), 7.47–7.50 (m, 2H), 7.88 (dd, *J* = 2.4 Hz, 6.0 Hz, 1H); ^{13}C NMR (100 MHz, $CDCl_3$) δ ppm 27.6, 36.1, 63.1, 121.1, 122.7, 123.6, 124.0, 125.4, 126.6, 128.5, 131.6, 132.0, 133.1, 143.3, 143.6, 144.1, 144.6, 168.4 (CO). EIMS *m*/*z* (relative intensity, %): 261 (100), 246 (15), 232 (31), 202 (20), 189 (5), 146 (92), 129 (6), 117 (14), 115 (15), 109 (6), 101 (15), 91 (14), 57 (4), 40 (27); HRMS–ESI (*m*/*z*) calcd for $C_{18}H_{15}NO+H^+$, 264.1383; found, 264.1385.

3-Cyclohexenyl-2-methylisoindolin-1-one (**3j**)

Colorless solid; mp 91–94 °C; ^{1}H NMR (400 MHz, $CDCl_3$) δ ppm 1.16–1.21 (m, 1H), 1.43–1.62 (m, 5H), 2.16 (d, *J* = 2.8 Hz, 2H), 3.00 (s, 3H), 4.74 (s, 1H), 6.07 (s, 1H), 7.32 (d, *J* = 7.6 Hz, 1H), 7.44 (t, *J* = 7.6 Hz, 1H), 7.51 (t, *J* = 6.8 Hz, 1H), 7.82 (d, *J* = 7.6 Hz, 1H); ^{13}C NMR (100 MHz, $CDCl_3$) δ ppm 21.7, 22.2, 22.4, 25.4, 27.0, 69.3, 122.3, 123.2, 128.1, 129.7, 131.3, 132.7, 133.9, 144.3, 168.6 (CO); EIMS *m*/*z* (relative intensity, %): 227 (76), 226 (23),

198 (20), 170 (12), 159 (7), 146 (100), 128 (6), 91 (20), 77 (5), 40 (12); HRMS–ESI (*m*/*z*) calcd for $C_{15}H_{17}NO+H^+$, 228.1383; found, 228.1379.

3-(3,4-Dihydro-2*H*-pyran-5-yl)-2-methylisoindolin-1-one (**3k**)
Colorless solid; mp 94–97 °C; ^{1}H NMR (400 MHz, $CDCl_3$) δ ppm 1.20–1.27 (m, 1H), 1.40–1.47 (m, 1H), 1.71–1.79 (m, 2H), 3.00 (s, 3H), 3.92–4.04 (m, 2H), 4.65 (s, 1H), 6.79 (s, 1H), 7.36 (d, *J* = 7.2 Hz, 1H), 7.44 (t, *J* = 7.2 Hz, 1H), 7.53 (t, *J* = 7.2 Hz, 1H), 7.81 (d, *J* = 7.2 Hz, 1H); ^{13}C NMR (100 MHz, $CDCl_3$) δ ppm 17.0, 21.5, 26.6, 65.2, 66.0, 108.2, 122.2, 123.0, 128.1, 131.3, 132.7, 144.2, 144.6, 168.2 (CO); EIMS *m/z* (relative intensity, %): 229 (100), 200 (47), 186 (35), 172 (54), 146 (51), 128 (20), 115 (17), 91 (24); HRMS–ESI (*m*/*z*) calcd for $C_{14}H_{15}NO_2+H^+$, 230.1176; found, 230.1175.

3-(4,5-Dihydrofuran-3-yl)-2-methylisoindolin-1-one (**3l**)
Pale yellow syrup; ^{1}H NMR (400 MHz, $CDCl_3$) δ ppm 1.91–2.00 (m, 1H), 2.12–2.20 (m, 1H), 3.05 (s, 3H), 4.34–4.40 (m, 2H), 5.11 (s, 1H), 6.71 (s, 1H), 7.37 (d, *J* = 7.6 Hz, 1H), 7.46 (t, *J* = 7.6 Hz, 1H), 7.54 (t, *J* = 7.6 Hz, 1H), 7.84 (d, *J* = 7.6 Hz, 1H); ^{13}C NMR (100 MHz, $CDCl_3$) δ ppm 27.0, 30.9, 59.2, 70.9, 110.3, 122.4, 123.4, 128.4, 131.5, 132.5, 143.8, 146.5, 168.2 (CO); EIMS *m/z* (relative intensity, %): 215 (64), 214 (40), 187 (63), 186 (100), 170 (57), 159 (43), 146 (51), 128 (21), 91 (21); HRMS–ESI (*m*/*z*) calcd for $C_{13}H_{13}NO_2+H^+$, 216.1019; found, 216.1017.

(*E*)-3-(2-Phenylethenyl)isoindolin-1-one (**3m**)
Colorless syrup; ^{1}H NMR (400 MHz, $CDCl_3$) δ ppm 5.21 (d, *J* = 8.8 Hz, 1H), 6.00 (dd, *J* = 8.8 Hz, 15.6 Hz, 1H), 6.83 (s, 1H), 6.84 (s, 1H), 7.31 (d, *J* = 7.2 Hz, 1H), 7.34–7.40 (m, 4H), 7.44 (d, *J* = 7.6 Hz, 1H), 7.51 (t, *J* = 7.2 Hz, 1H), 7.59 (dt, *J* = 1.2 Hz, 7.2 Hz, 1H), 7.89 (d, *J* = 7.6 Hz, 1H); ^{13}C NMR (100 MHz, $CDCl_3$) δ ppm 59.6, 123.4, 123.9, 126.1, 126.6 (2C), 128.4, 128.6 (2C), 128.7, 131.3, 132.3, 133.9, 135.8, 146.4, 170.8 (CO); EIMS *m/z* (relative intensity, %): 235 (30), 206 (15), 182 (15), 168 (19), 144 (64) 44 (100); HRMS–ESI (*m*/*z*) calcd for $C_{16}H_{13}NO+H^+$, 236.1070; found, 236.1068.

3-(2-Phenyl-2-propenyl)isoindolin-1-one (**4d**)
Pale yellow syrup; ^{1}H NMR (400 MHz, $CDCl_3$) δ ppm 2.68 (dd, *J* = 9.2 Hz, 14.0 Hz, 1H), 3.17 (dd, *J* = 4.8 Hz, 14.0 Hz, 1H), 4.63 (dd, *J* = 4.8 Hz, 9.2 Hz, 1H), 5.19 (s, 1H), 5.45 (s, 1H), 7.00 (s, 1H), 7.31 (d, *J* = 7.6 Hz, 1H), 7.37 (dt, *J* = 0.8 Hz, 7.6 Hz, 2H), 7.46 (t, *J* = 6.8 Hz. 4H), 7.54 (t, *J* = 7.6 Hz, 1H), 7.84 (d, *J* = 7.6 Hz, 1H); ^{13}C NMR (100 MHz, $CDCl_3$) δ ppm 41.3, 55.0, 116.1, 122.6, 123.7, 126.2 (2C), 128.0, 128.2, 128.6 (2C), 131.6, 131.8, 139.8, 144.5, 147.0, 170.3 (CO); EIMS *m/z* (relative intensity, %): 249 (4), 201 (2), 174 (2), 149 (6), 132 (100), 115 (7), 104 (10), 77 (10), 44 (23); HRMS–ESI (*m*/*z*) calcd for $C_{17}H_{15}NO+H^+$, 250.1227; found, 250.1223.

3-(2,2-Diphenylethenyl)isoindolin-1-one (**3n**)
Colorless solid; mp 205–207 °C; ^{1}H NMR (400 MHz, $CDCl_3$) δ ppm 5.22 (d, *J* = 9,6 Hz, 1H), 5.82 (d, *J* = 9,6 Hz, 1H), 6.97 (s, 1H), 7.21–7.27 (m, 4H), 7.38–7.49 (m, 8H), 7.57 (dt, *J* = 0.8 Hz, 7.6 Hz, 1H), 7.86 (d, *J* = 7.6 Hz, 1H); ^{13}C NMR (100 MHz, $CDCl_3$) δ ppm 56.0, 123.3,

123.8, 124.9, 127.4 (2C), 127.9, 128.1, 128.3 (2C), 128.4, 128.8 (2C), 129.7 (2C), 131.6, 132.0, 138.6, 140.8, 146.7, 146.9, 170.7 (CO); EIMS *m/z* (relative intensity, %): 311 (6), 306 (2), 276 (3), 215 (18), 200 (40), 172 (51), 149 (68), 129 (16), 97 (27) 44 (100); HRMS–ESI (*m*/*z*) calcd for $C_{22}H_{17}NO+H^+$, 312.1383; found, 312.1386.

3-(1*H*-Inden-2-yl)isoindolin-1-one (**3o**)

Pale yellow solid; mp 201–204 °C; ^{1}H NMR (400 MHz, $CDCl_3$) δ ppm 2.96 (d, *J* = 22.8 Hz, 1H), 3.35 (d, *J* = 22.8 Hz, 1H), 5.66 (s, 1H), 6.66 (s, 1H), 7.03 (s, 1H), 7.17 (dt, *J* = 1.2 Hz, 7.6 Hz, 1H), 7.27 (t, *J* = 7.2 Hz, 1H), 7.33 (d, *J* = 7.6 Hz, 2H), 7.37 (t, *J* = 7.6 Hz, 1H), 7.49 (d, *J* = 7.2 Hz, 1H), 7.51–7.57 (m, 1H), 7.90 (d, *J* = 7.6 Hz, 1H); ^{13}C NMR (100 MHz, $CDCl_3$) δ ppm 36.5, 57.4, 121.1, 123.2, 123.9, 124.0, 125.3, 126.6, 128.6, 131.0, 131.1, 132.3, 143.2, 143.7, 145.1, 146.6, 170.6 (CO); EIMS *m/z* (relative intensity, %): 247 (25), 218 (12), 194 (8), 180 (10), 165 (9), 149 (20), 132 (23), 129 (31), 111 (18), 83 (24), 57 (36), 44 (100); HRMS–ESI (*m*/*z*) calcd for $C_{17}H_{13}NO+H^+$, 248.1070; found, 248.1073.

(*E*)-1-Benzyl-5-(2-phenylethenyl)-1*H*-pyrrol-2(5*H*)-one (**6a**)

Colorless syrup; ^{1}H NMR (400 MHz, $CDCl_3$) δ ppm 4.08 (d, *J* = 14.8 Hz, 1H), 4.54 (d, *J* = 9.2 Hz, 1H), 5.12 (d, *J* = 14.8 Hz, 1H), 5.69 (dd, *J* = 9.2 Hz, 15.6 Hz, 1H), 6.26 (dd, *J* = 1.6 Hz, 5.6 Hz, 1H), 6.59 (d, *J* = 15.6 Hz, 1H), 6.96 (dd, *J* = 1.6 Hz, 6.0 Hz, 1H), 7.23–7.35 (m, 8H), 7.40 (dd, *J* = 1.6 Hz, 8.0 Hz, 2H); ^{13}C NMR (100 MHz, $CDCl_3$) δ ppm 42.4, 64.8, 126.1, 126.6, 127.4, 128.0, 128.2, 128.6 (2C), 128.7 (2C), 128.7 (2C), 128.9 (2C), 135.7, 137.6, 146.6, 170.9 (CO); EIMS *m/z* (relative intensity, %): 275 (22), 190 (11), 189 (100), 184 (30), 161 (29), 160 (39), 132 (37), 119 (22), 104 (48), 91 (21); HRMS–ESI (*m*/*z*) calcd for $C_{19}H_{17}NO+H^+$, 276.1383; found, 276.1385.

1-Benzyl-5-(2-phenyl-2-propenyl)-1*H*-pyrrol-2(5*H*)-one (**7a**)

Brown syrup; ^{1}H NMR (400 MHz, $CDCl_3$) δ ppm 2.34 (dd, *J* = 10.4 Hz, 13.6 Hz, 1H), 3.20 (dd, *J* = 4.4 Hz, 13.6 Hz, 1H), 3.96–4.00 (m, 1H), 4.16 (d, *J* = 15.2 Hz, 1H), 5.08 (s, 1H), 5.15 (d, *J* = 15.2 Hz, 1H), 5.38 (s, 1H), 6.12 (dd, *J* = 1.6 Hz, 6.0 Hz, 1H), 6.91 (dd, *J* = 1.6 Hz, 6.0 Hz, 1H), 7.19–7.26 (m, 7H), 7.28–7.31 (m, 3H); ^{13}C NMR (100 MHz, $CDCl_3$) δ ppm 37.0, 43.7, 60.5, 115.9, 125.8 (2C), 126.5, 127.5, 127.8 (2C), 127.9 (2C), 128.5, 128.7 (2C), 137.2, 139.6, 143.4, 147.8, 171.0 (CO); EIMS *m/z* (relative intensity, %): 289 (17), 274 (6), 220 (17), 172 (48), 171 (35), 91 (100), 40 (58); HRMS–ESI (*m*/*z*) calcd for $C_{20}H_{19}NO+H^+$, 290.1540; found, 290.1508.

1-Benzyl-5-(2,2-diphenylethenyl)-1*H*-pyrrol-2(5*H*)-one (**6b**)

Brown syrup; ^{1}H NMR (400 MHz, $CDCl_3$) δ ppm 4.25 (d, *J* = 14.8 Hz, 1H), 4.64–4.67 (m, 1H), 4.88 (d, *J* = 14.8 Hz, 1H), 5.49 (d, *J* = 9.6 Hz, 1H), 6.23 (dd, *J* = 1.6 Hz, 6.0 Hz, 1H), 6.94 (dd, *J* = 1.6 Hz, 6.0 Hz, 1H), 7.03–7.10 (m, 6H), 7.20–7.21 (m, 3H), 7.25–7.26 (m, 3H), 7.28–7.30 (m, 3H); ^{13}C NMR (100 MHz, $CDCl_3$) δ ppm 44.2, 62.0, 122.8, 127.2, 127.3, 127.4 (2C), 127.9, 128.0 (2C), 128.1 (2C), 128.2 (2C), 128.4 (2C), 128.5, 129.3 (2C), 137.5, 138.3, 140.7, 146.9, 147.7, 171.0 (CO); EIMS *m/z* (relative intensity, %): 351 (2), 190 (12), 189 (100), 182 (8), 161 (28), 160 (43), 132 (37), 129 (18), 119 (22), 104 (46), 91 (21), 77 (17); HRMS–ESI (*m*/*z*) calcd for $C_{25}H_{21}NO+H^+$, 352.4476; found, 352.4473.

1-Methyl-5-(2-phenyl-2-propenyl)-1*H*-pyrrol-2(5*H*)-one (7b)

Brown syrup; ^{1}H NMR (400 MHz, $CDCl_3$) δ ppm 2.51 (dd, *J* = 9.2 Hz, 14.0 Hz, 1H), 2.97 (s, 3H), 3.17 (dd, *J* = 4.8 Hz, 14.0 Hz, 1H), 4.04–4.07 (m, 1H), 5.17 (s, 1H), 5.51 (s, 1H), 6.08 (dd, *J* = 1.6 Hz, 6.0 Hz, 1H), 6.91 (dd, *J* = 1.6 Hz, 6.0 Hz, 1H), 7.34–7.40 (m, 5H); ^{13}C NMR (100 MHz, $CDCl_3$) δ ppm 27.2, 37.3, 63.3, 116.2, 126.1 (2C), 127.1, 128.0, 128.7 (2C), 140.2, 143.8, 147.2, 171.2 (CO); EIMS *m/z* (relative intensity, %): 213 (41), 198 (11), 154 (4), 115 (11), 96 (100), 91 (3), 77 (5); HRMS–ESI (*m*/*z*) calcd for $C_{14}H_{15}NO+H^+$, 214.1227; found, 214.1228.

5-(2,2-Diphenylethenyl)-1-methyl-1*H*-pyrrol-2(5*H*)-one (6c)

Brown syrup; ^{1}H NMR (400 MHz, $CDCl_3$) δ ppm 2.92 (s, 3H), 4.63 (d, *J* = 10.0 Hz, 1H), 5.57 (d, *J* = 10.0 Hz, 1H), 6.19 (dd, *J* = 1.6 Hz, 6.0 Hz, 1H), 6.94 (dd, *J* = 1.6 Hz, 6.0 Hz, 1H), 7.22–7.31 (m, 7H), 7.40–7.45 (m, 3H); ^{13}C NMR (100 MHz, $CDCl_3$) δ ppm 27.1, 63.8, 122.7, 127.2 (2C), 127.8, 128.0, 128.2 (2C), 128.4, 128.7 (2C), 129.4 (2C), 138.5, 140.5, 146.1, 148.1, 171.0 (CO); EIMS *m/z* (relative intensity, %): 275 (34), 260 (19), 114 (14), 113 (100), 161 (21), 91 (8); HRMS–ESI (*m*/*z*) calcd for $C_{19}H_{17}NO+H^+$, 276.1383; found, 276.1379.

^{1}H NMR and ^{13}C NMR spectra of **3a**

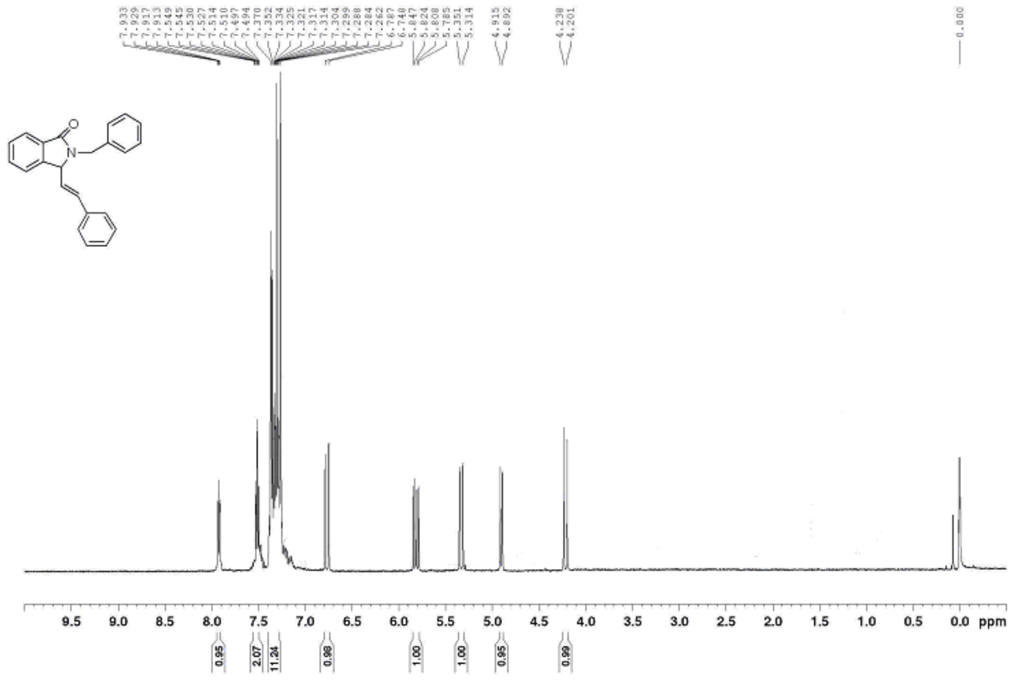

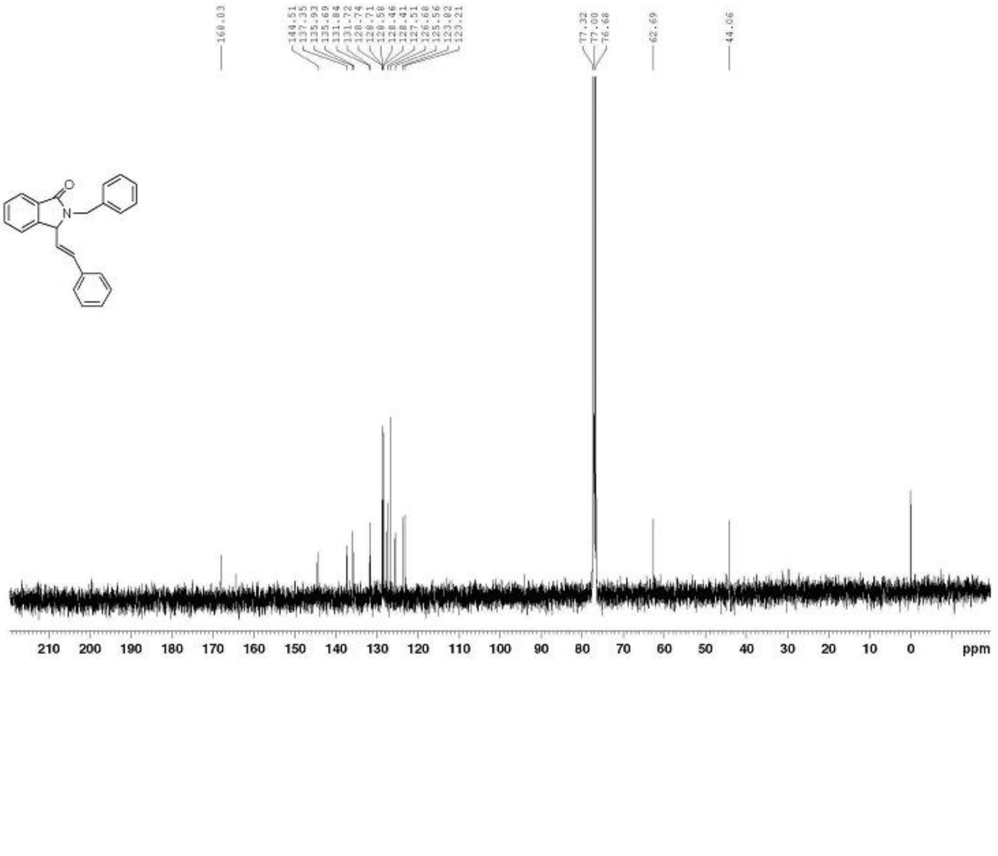

^{1}H NMR and ^{13}C NMR spectra of **4a**

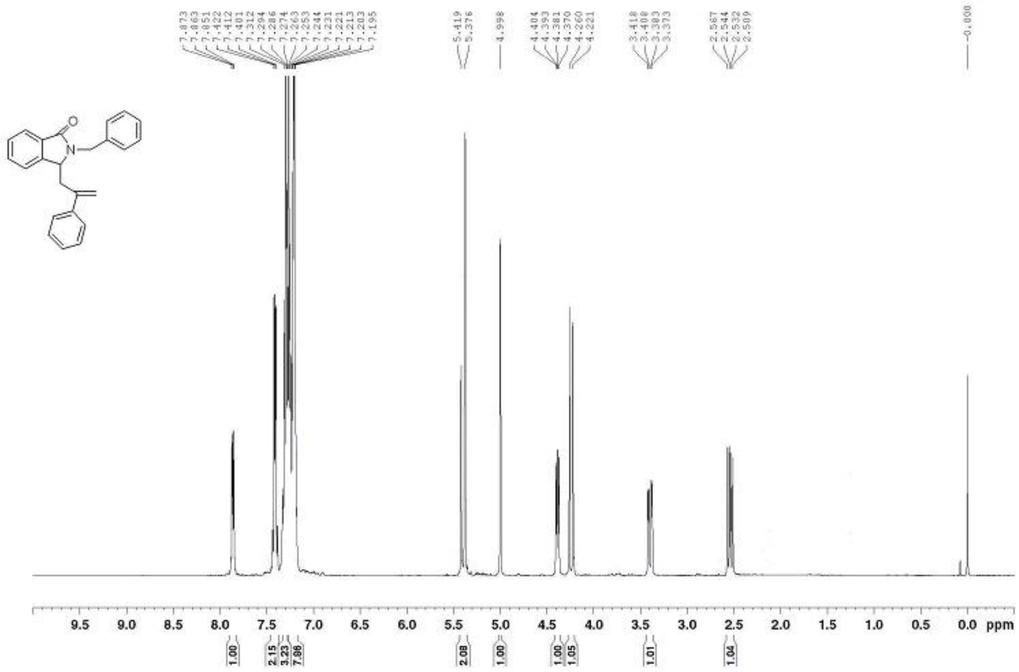

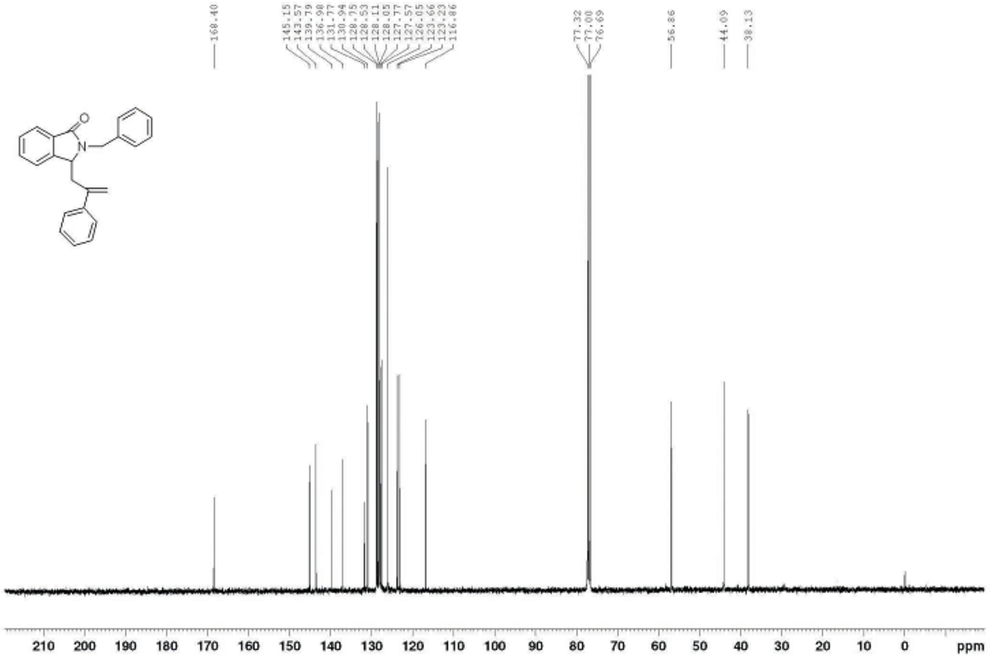

^{1}H NMR and ^{13}C NMR spectra of **3b**

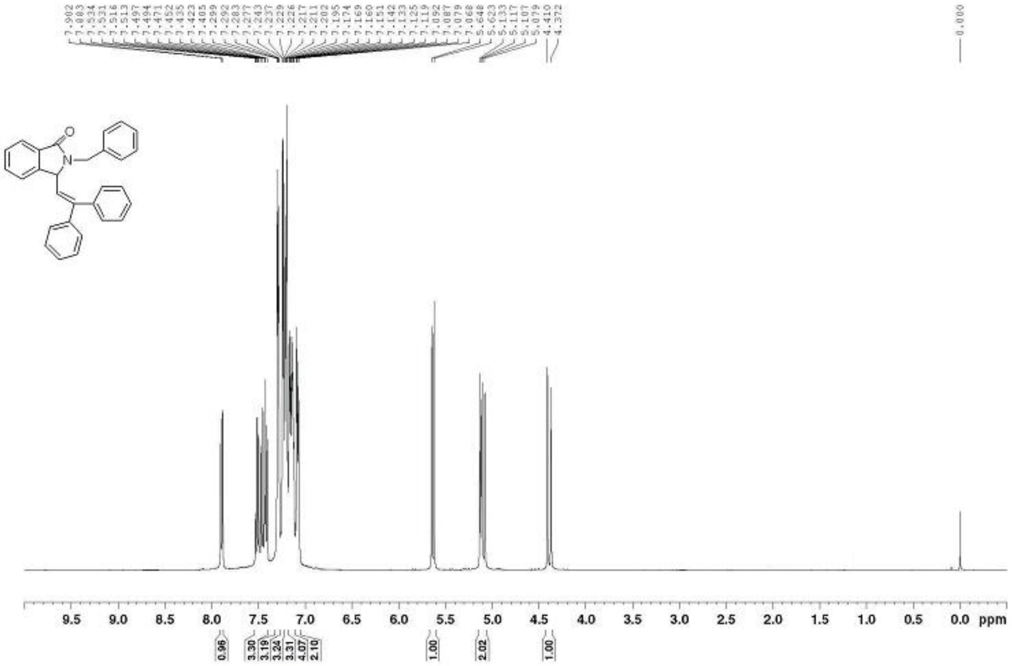

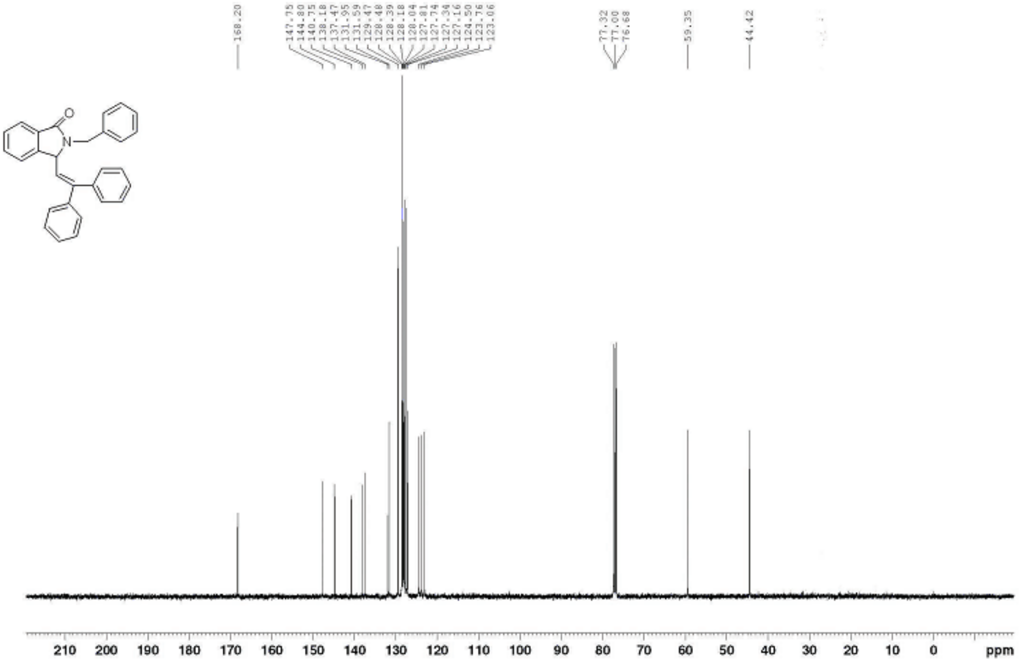

^{1}H NMR and ^{13}C NMR spectra of **3c**

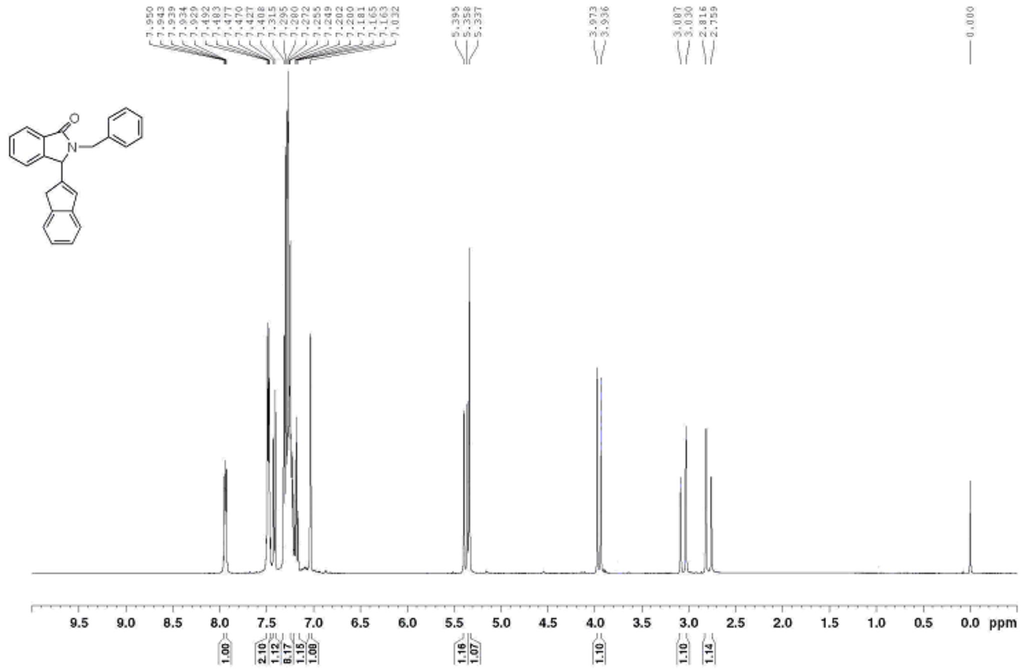

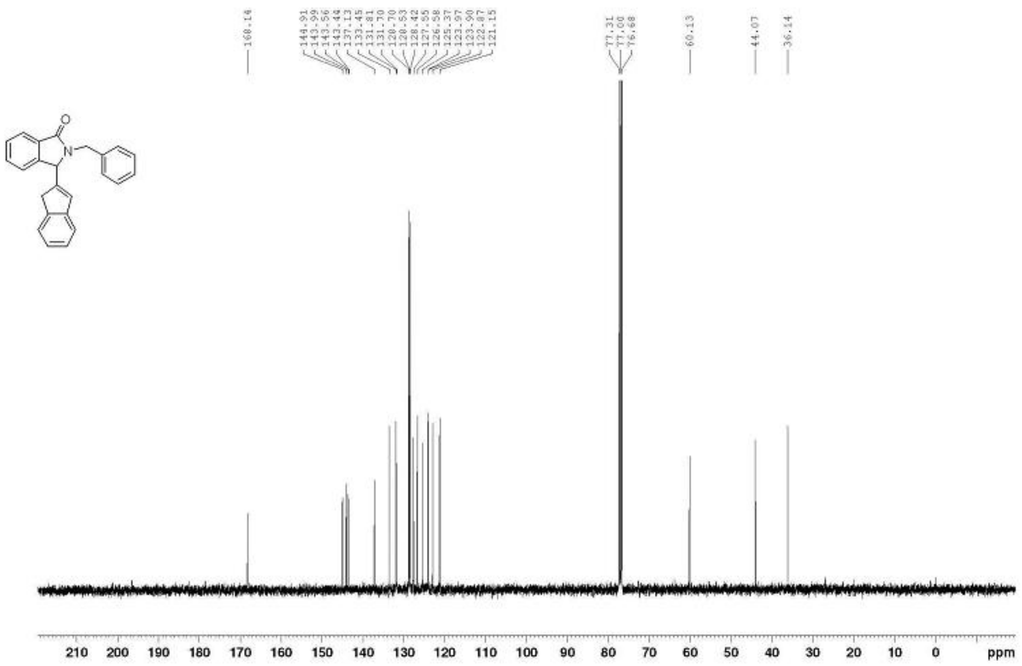

^{1}H NMR and ^{13}C NMR spectra of **3d**

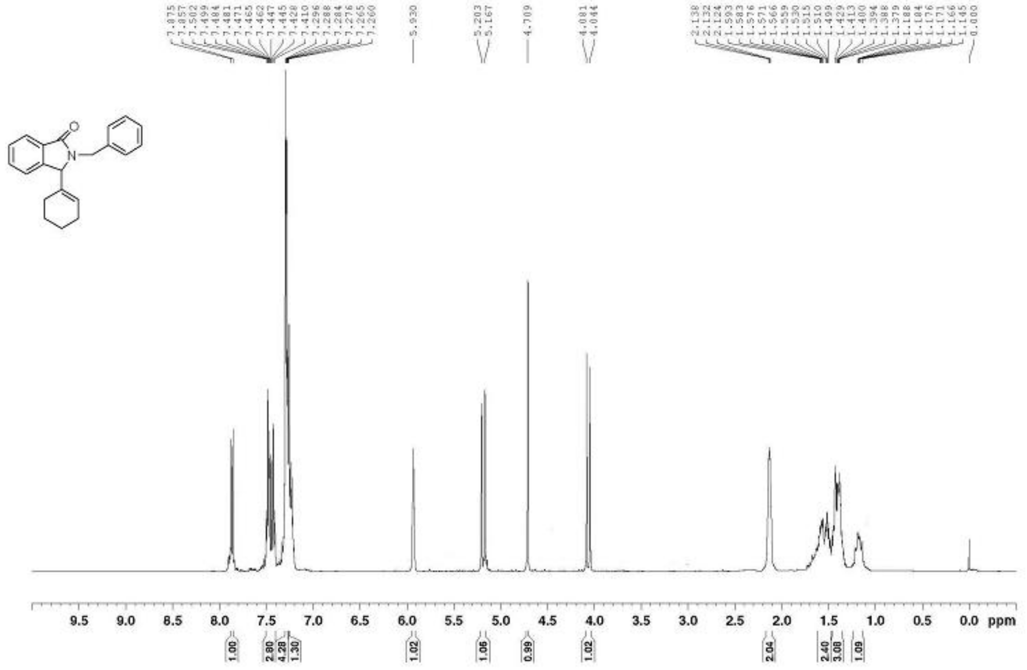

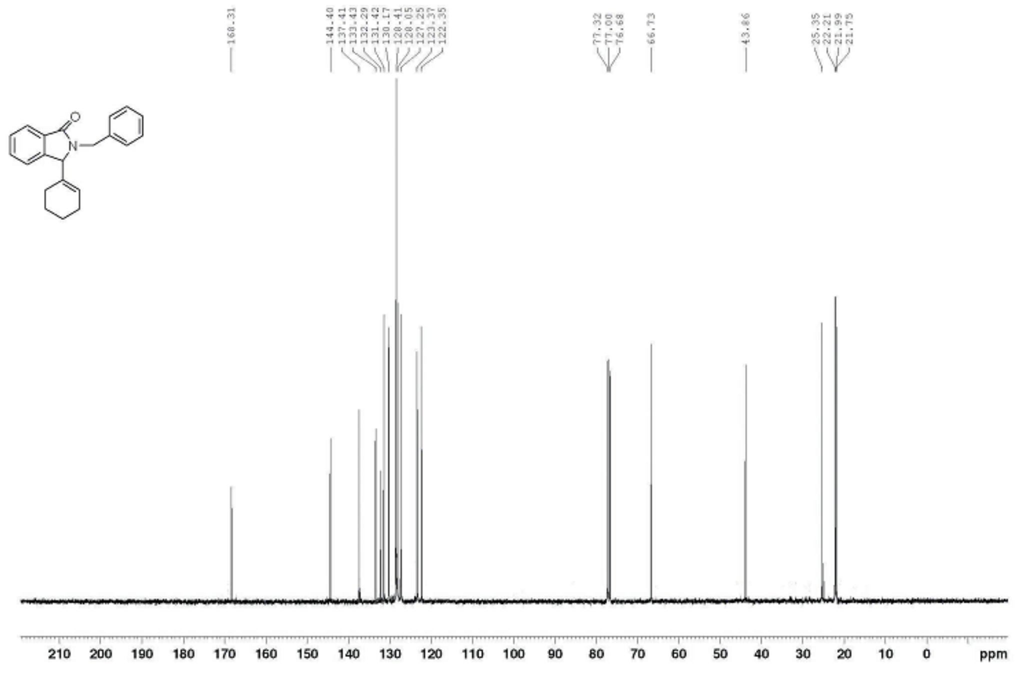

^{1}H NMR and ^{13}C NMR spectra of **3e**

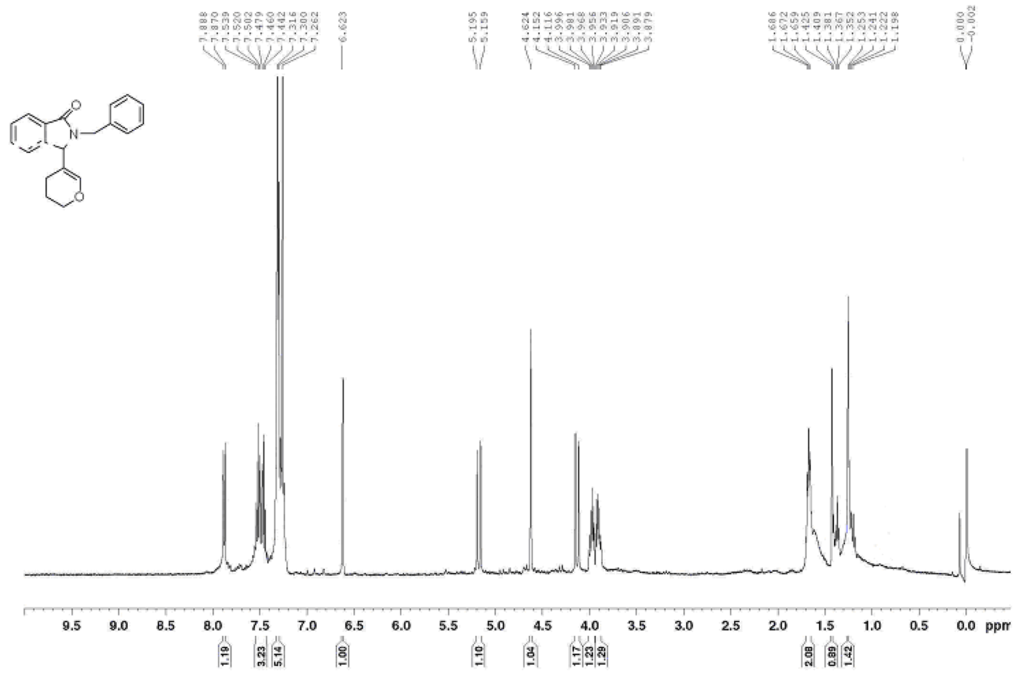

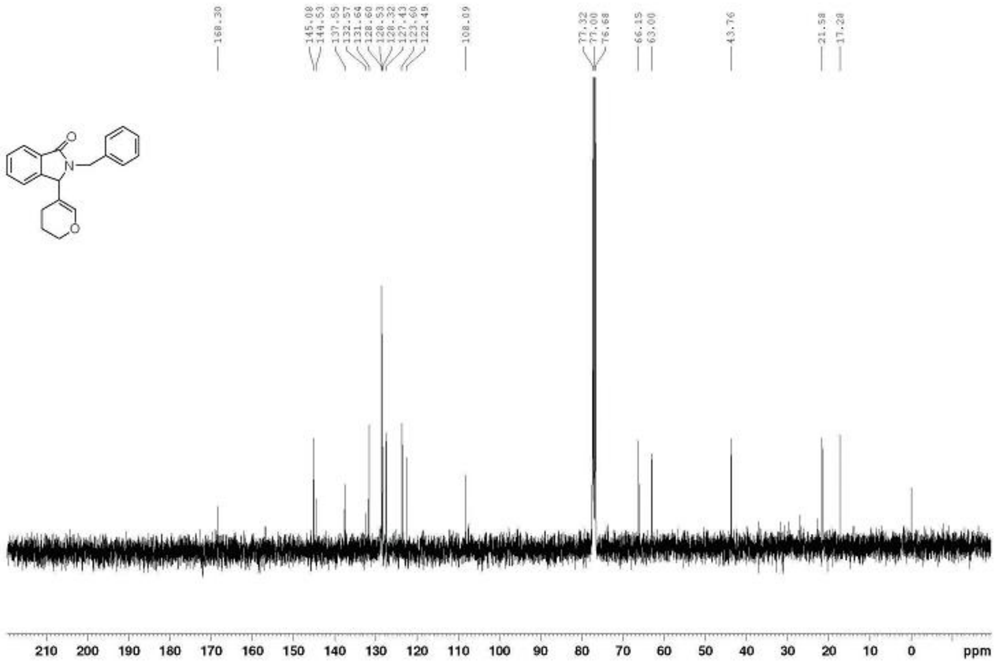

^{1}H NMR and ^{13}C NMR spectra of **3f**

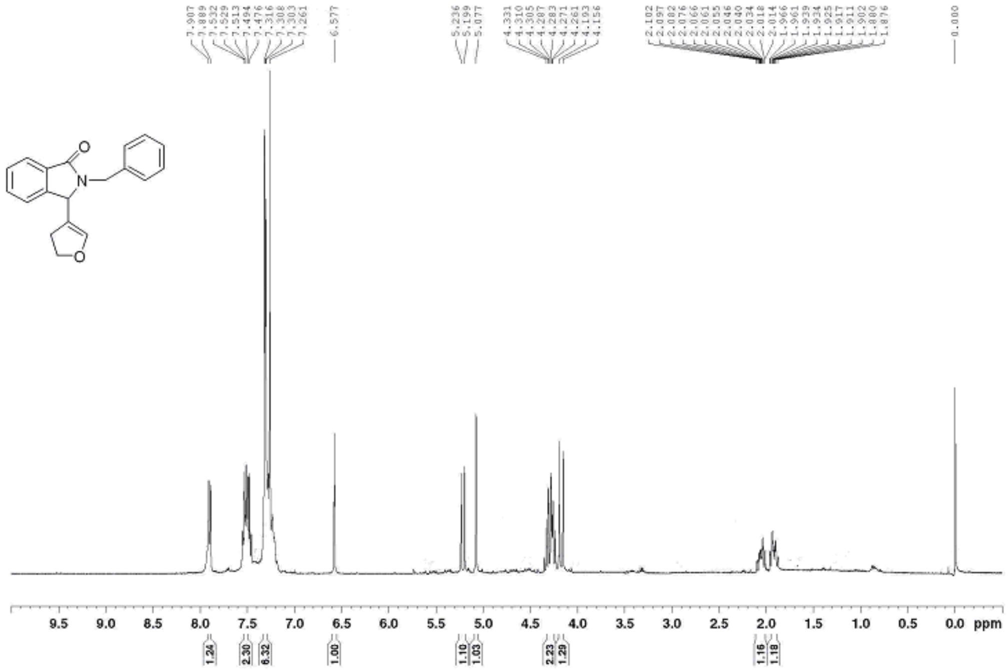

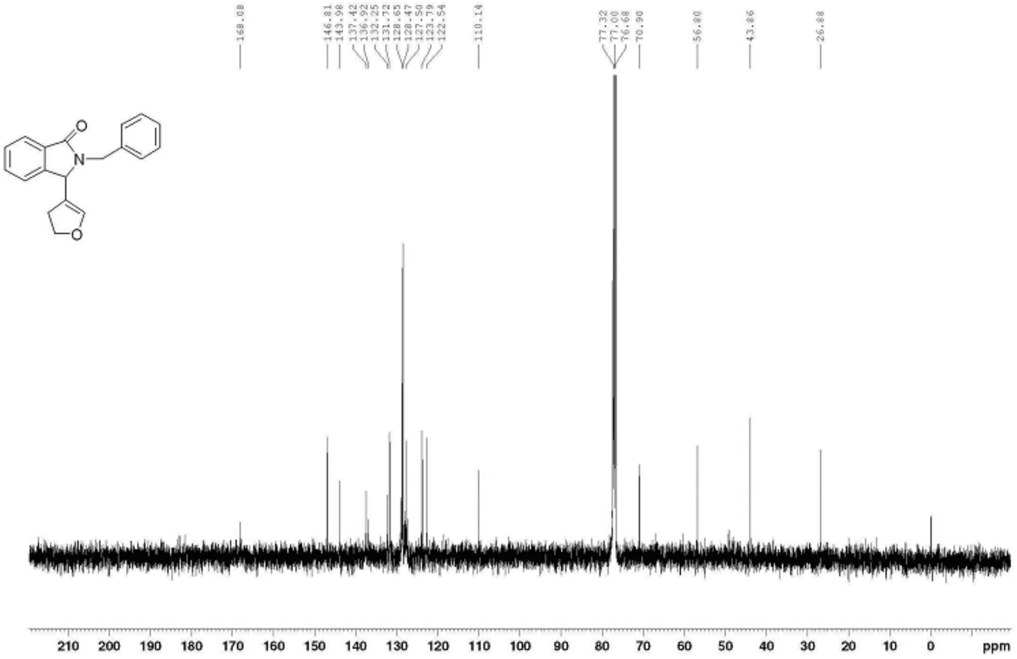

^{1}H NMR and ^{13}C NMR spectra of **4b**

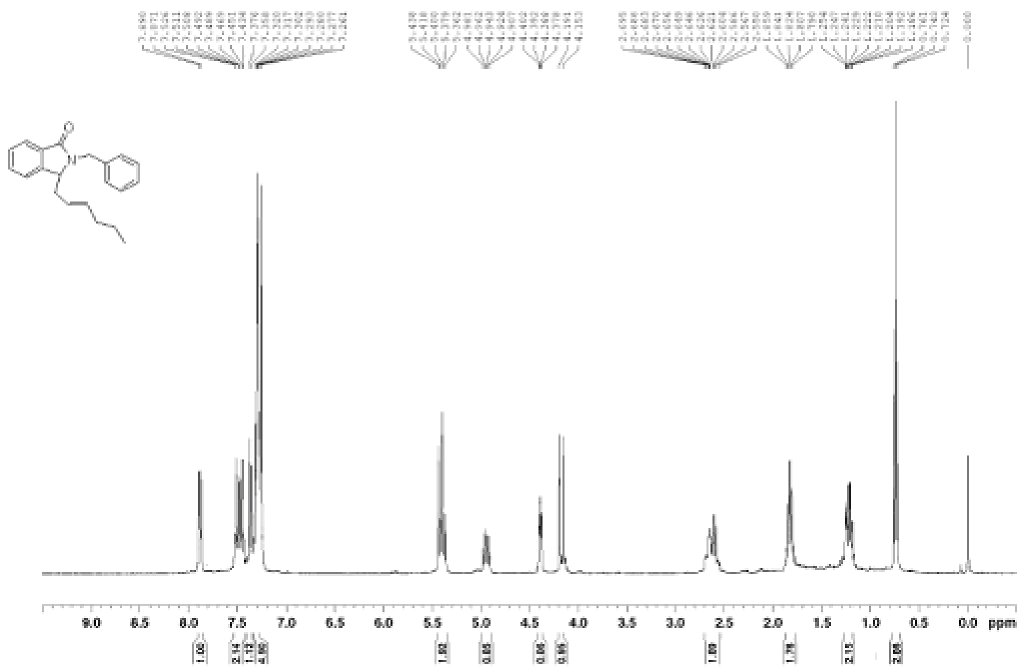

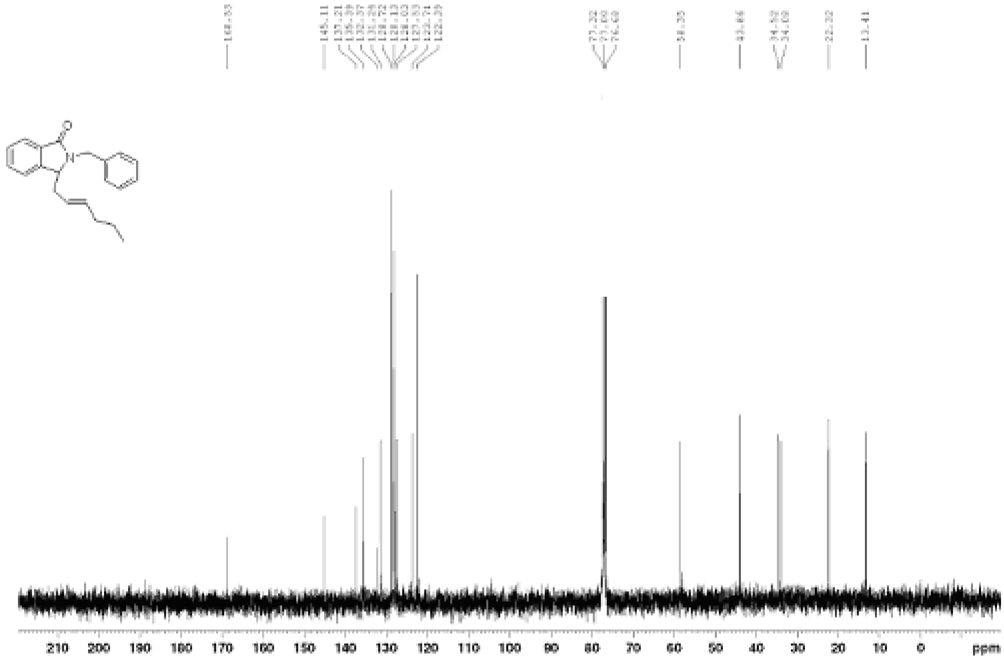

^{1}H NMR and ^{13}C NMR spectra of **3g**

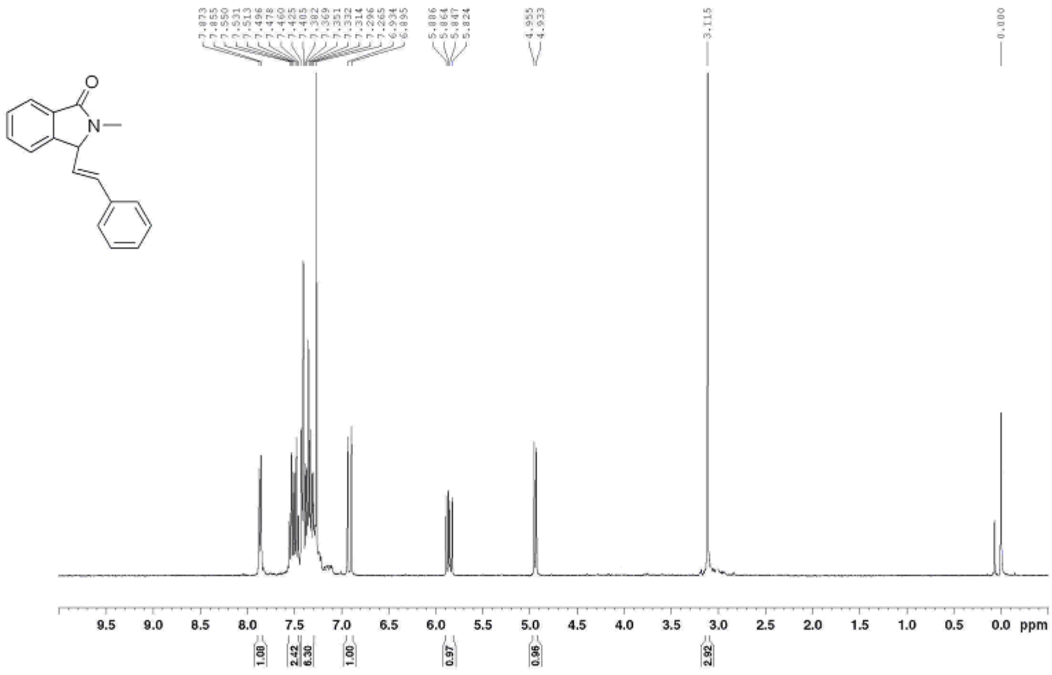

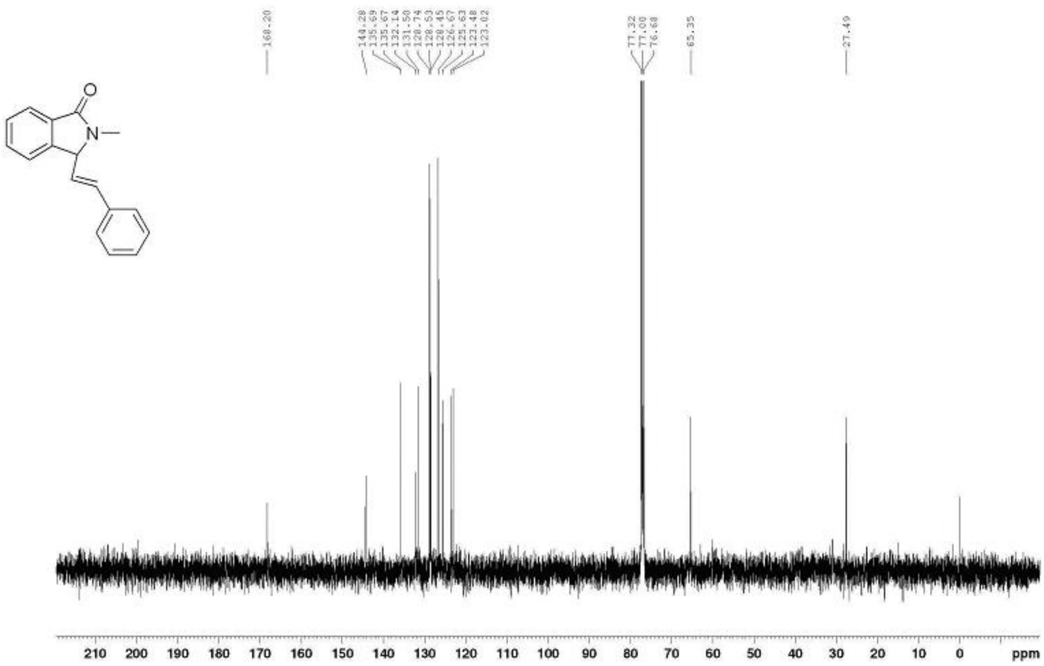

^{1}H NMR and ^{13}C NMR spectra of **4c**

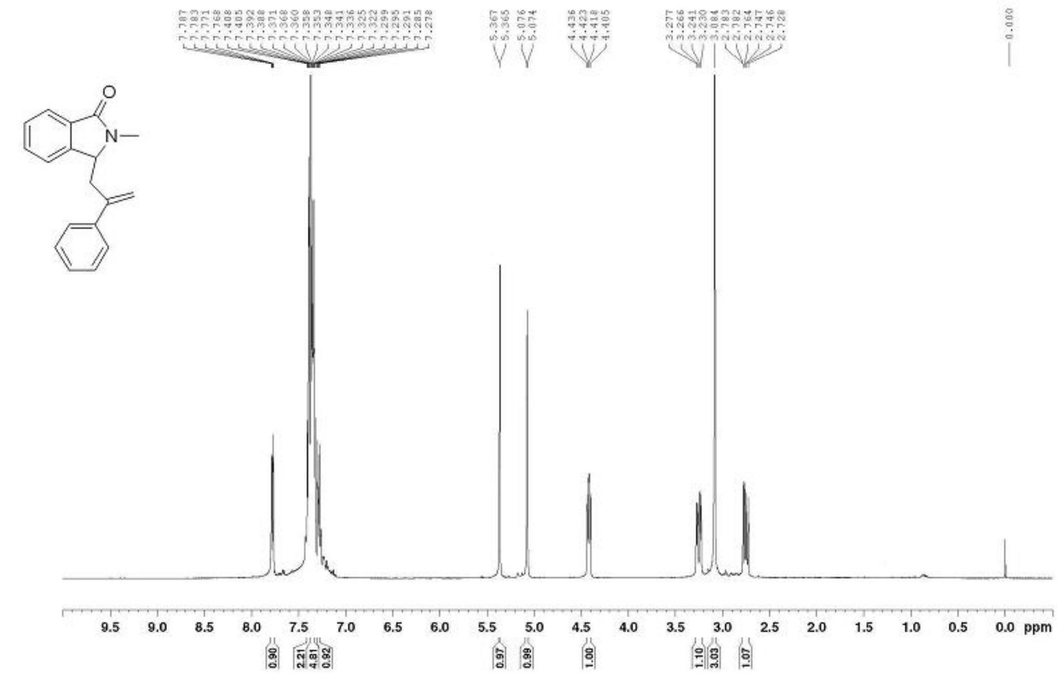

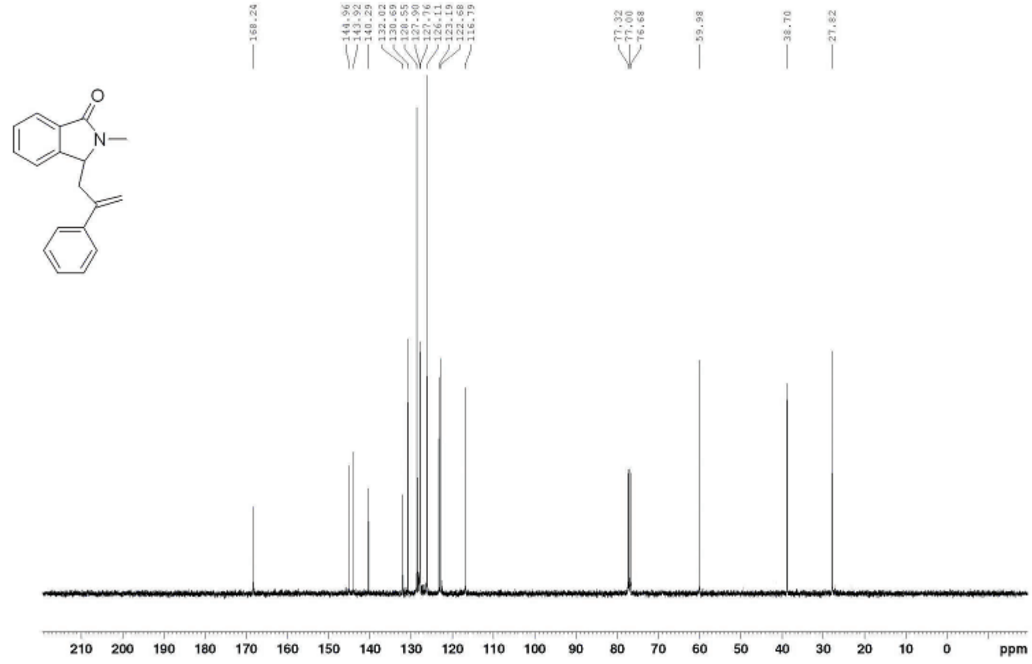

^{1}H NMR and ^{13}C NMR spectra of **3h**

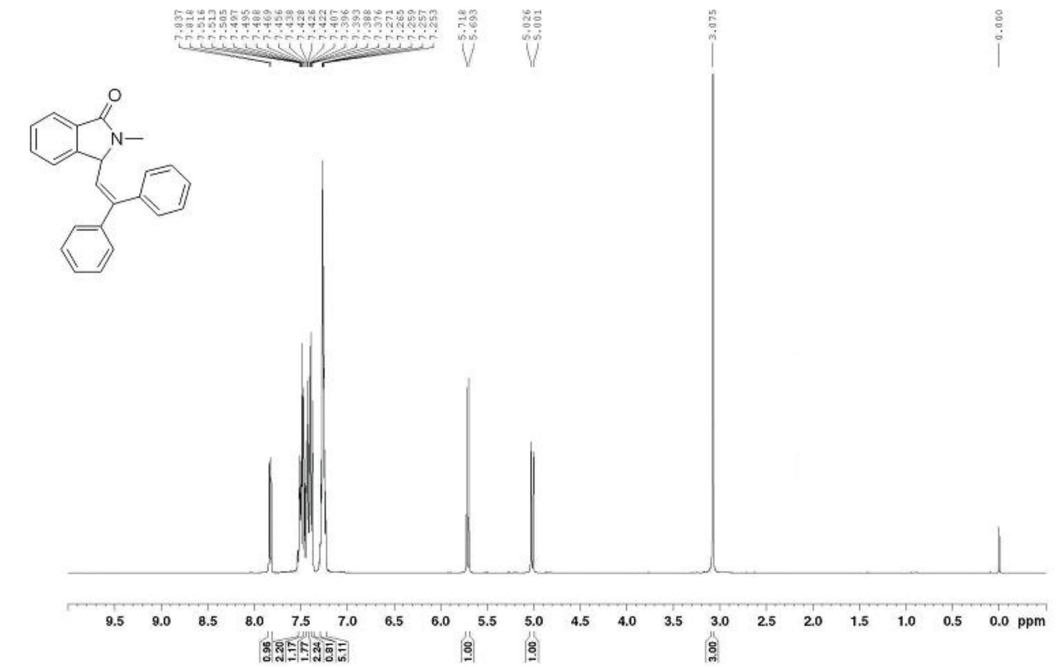

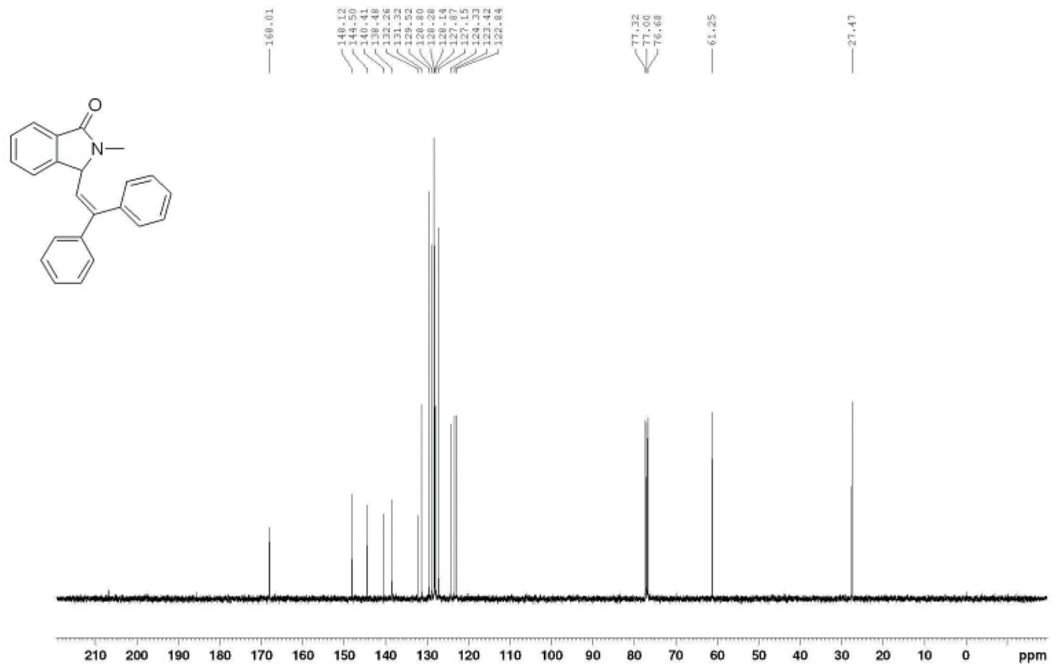

^{1}H NMR and ^{13}C NMR spectra of **3i**

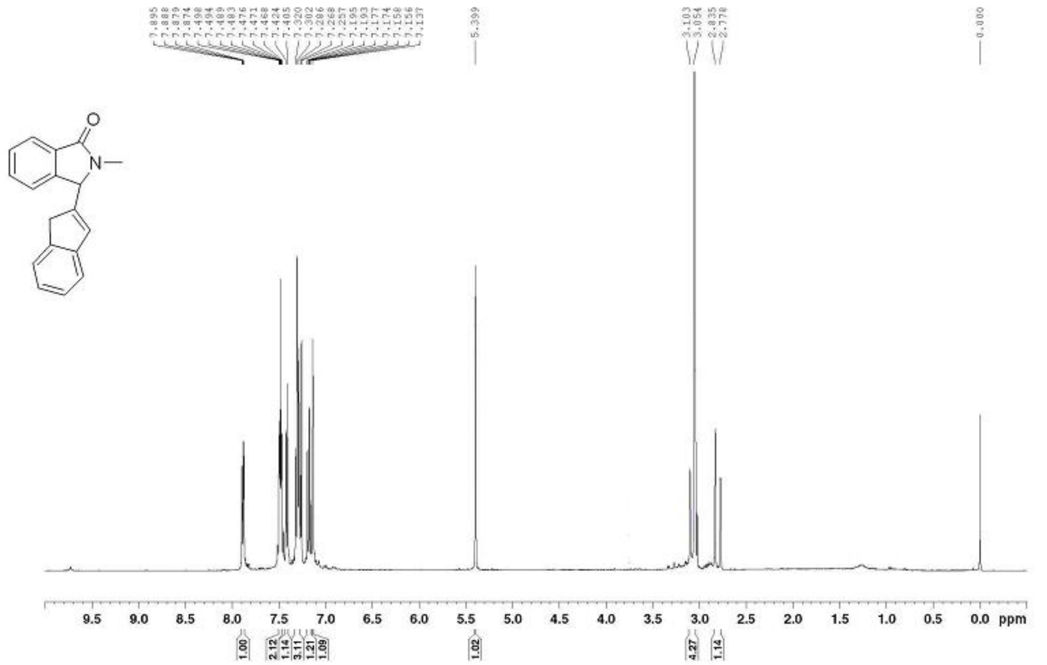

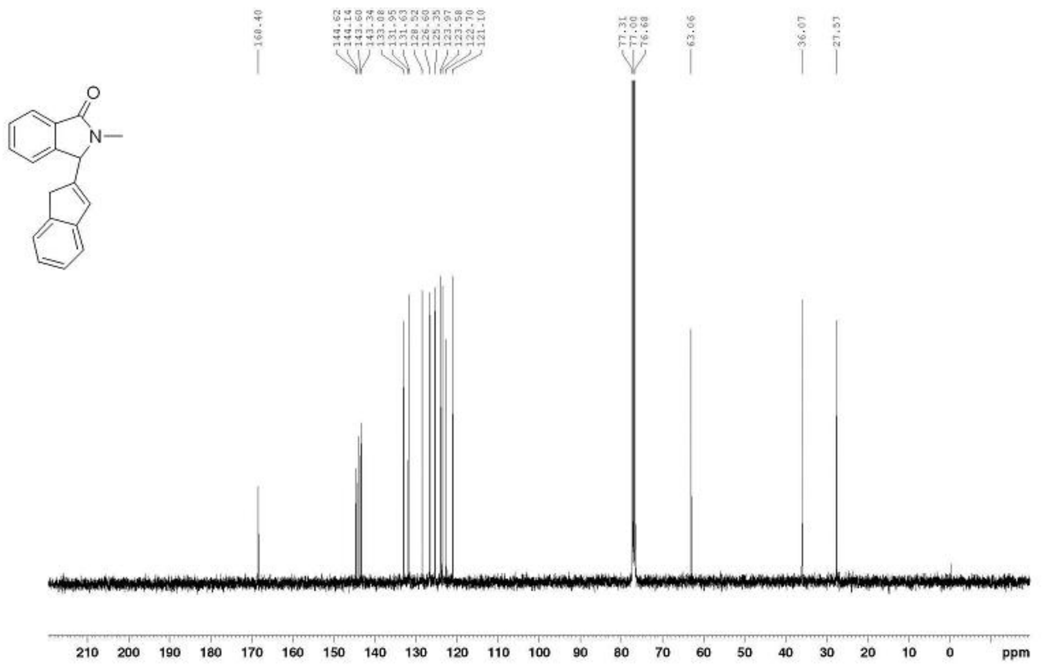

^{1}H NMR and ^{13}C NMR spectra of **3j**

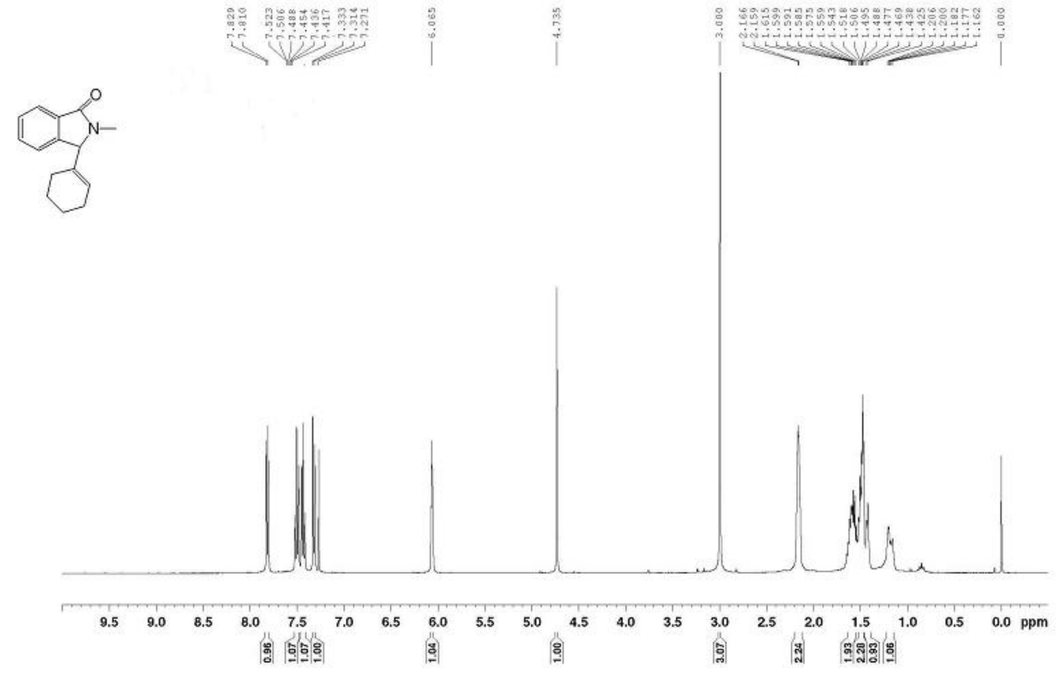

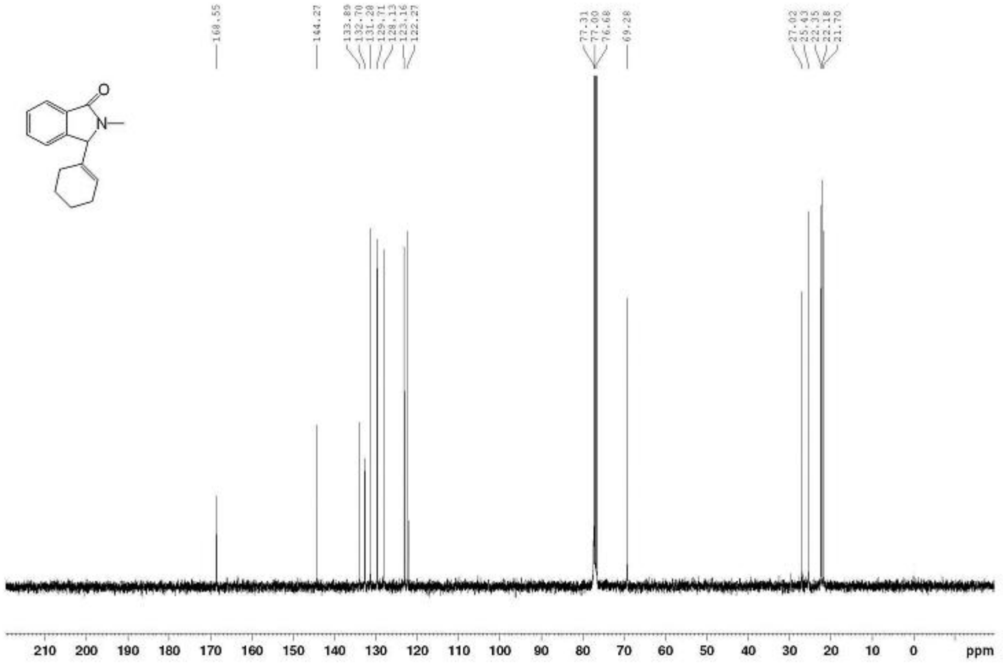

^{1}H NMR and ^{13}C NMR spectra of **3k**

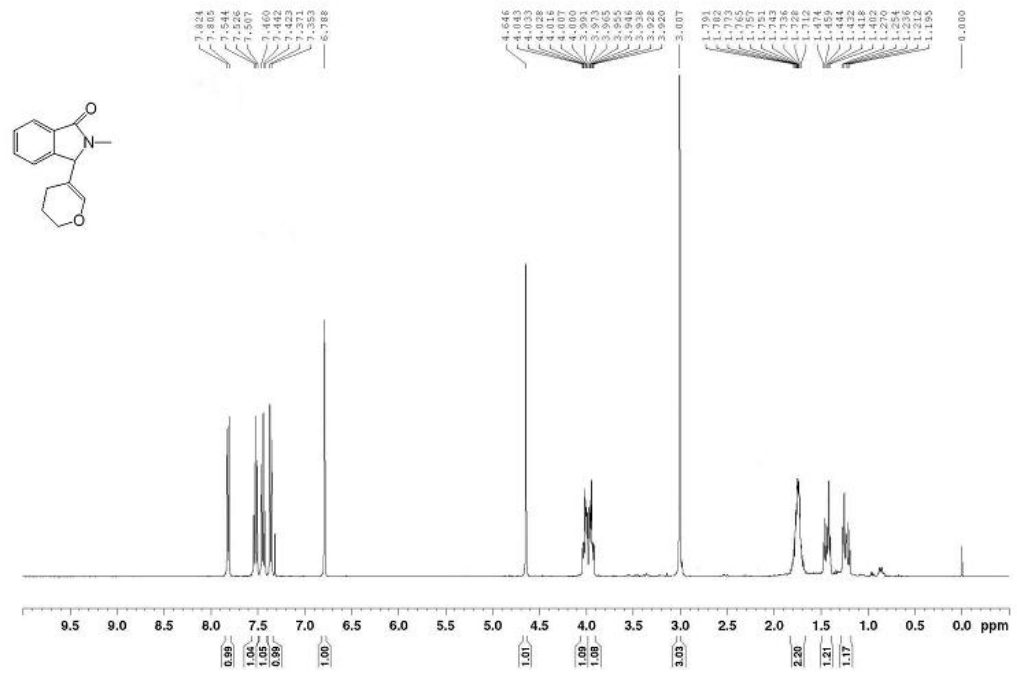

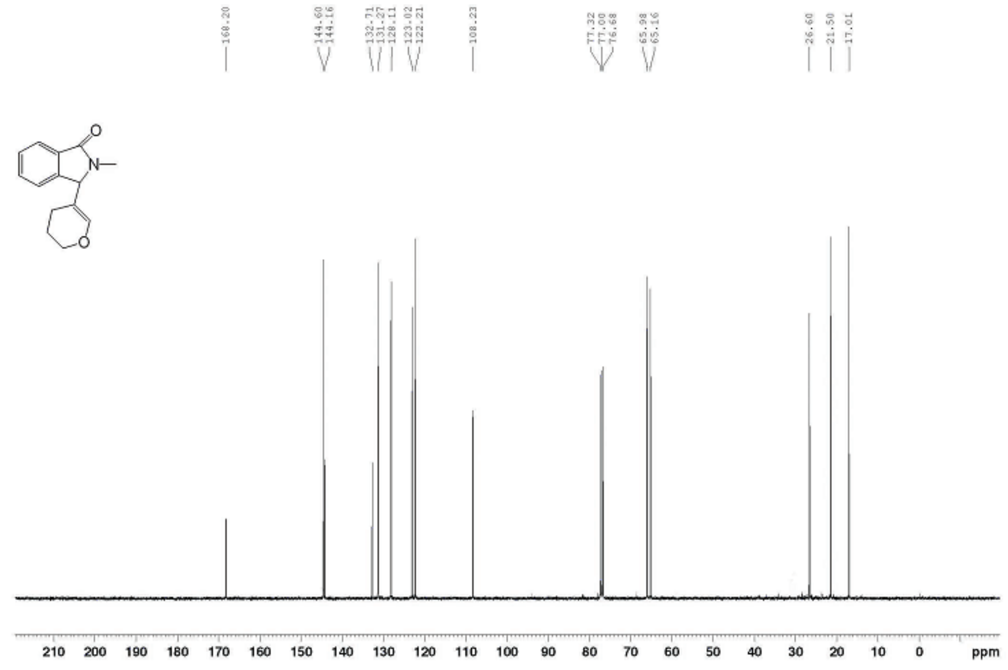

^{1}H NMR and ^{13}C NMR spectra of **3l**

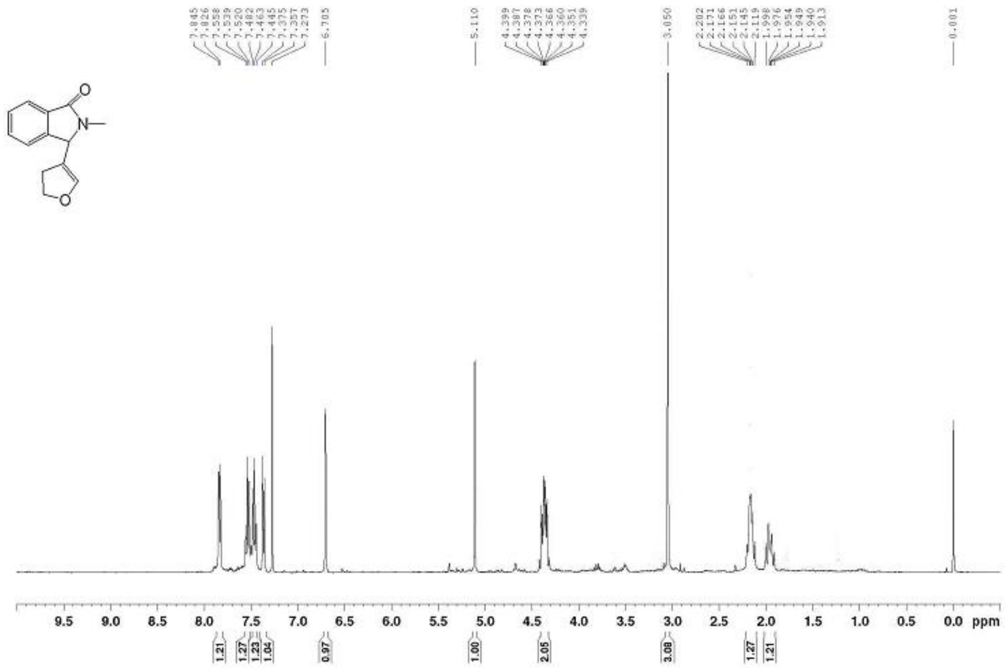

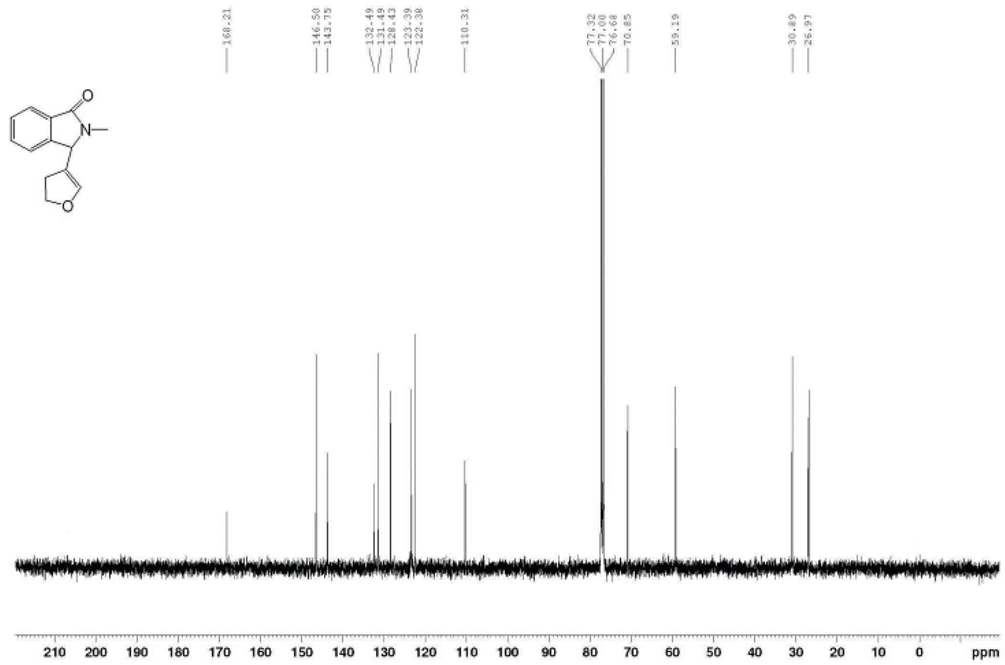

^{1}H NMR and ^{13}C NMR spectra of **3m**

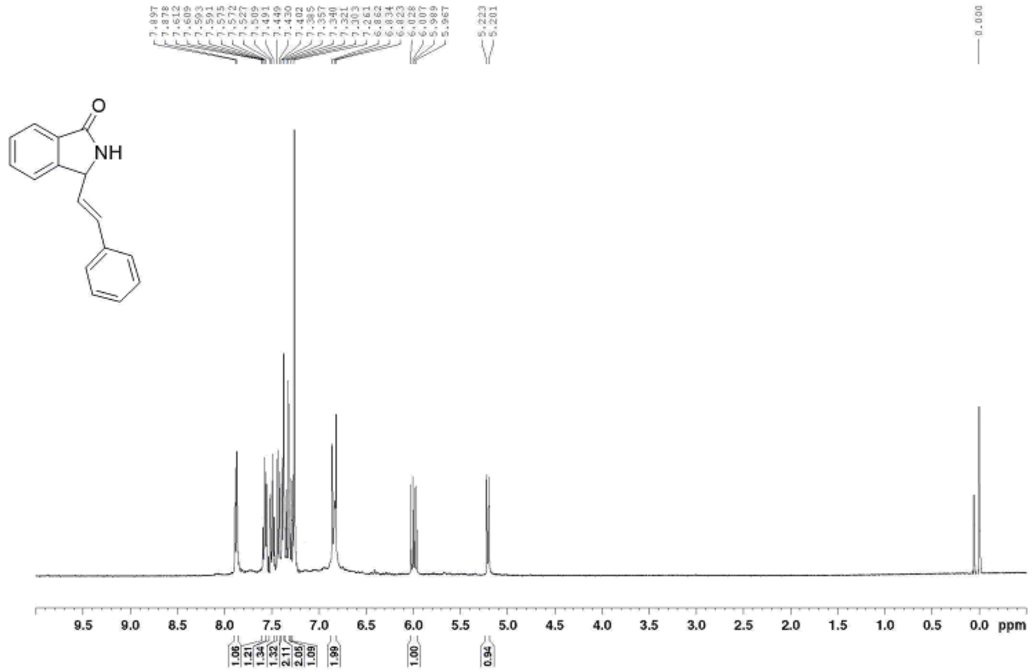

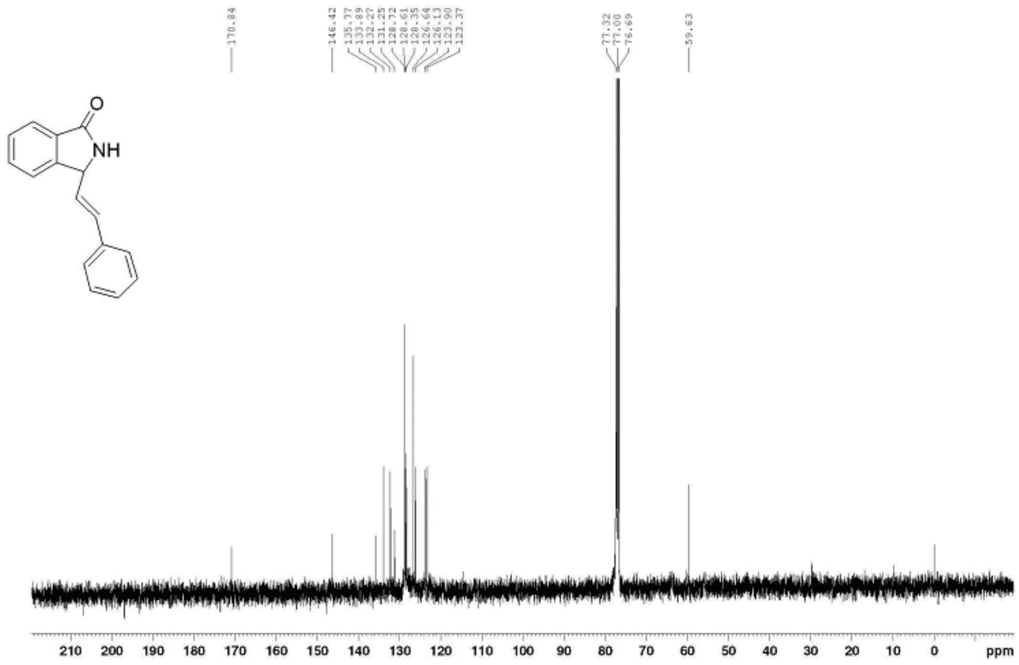

^{1}H NMR and ^{13}C NMR spectra of **4d**

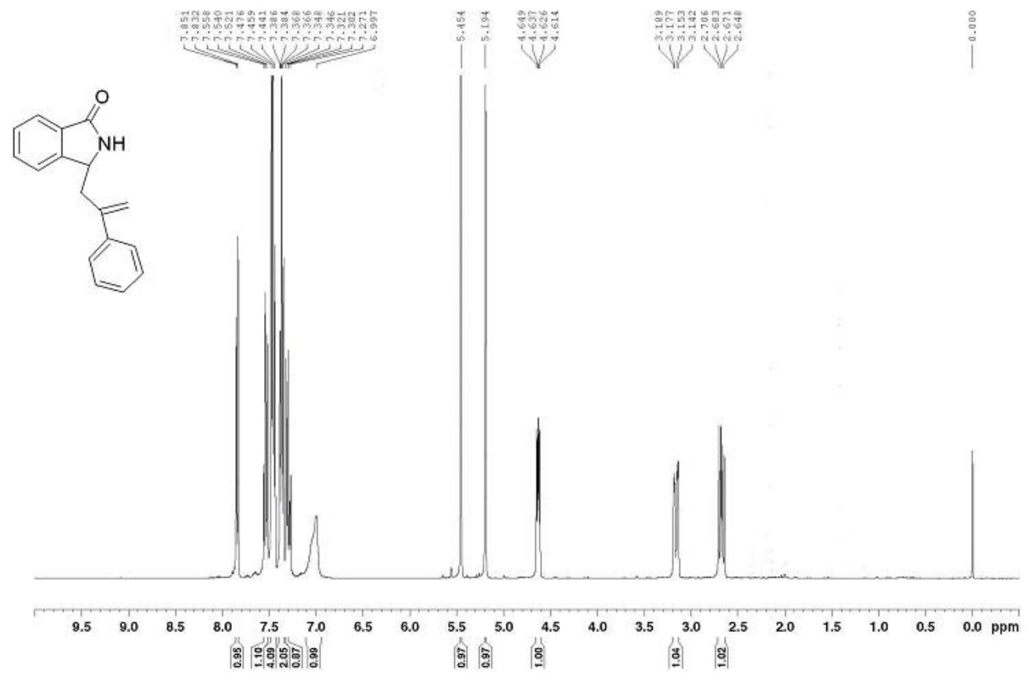

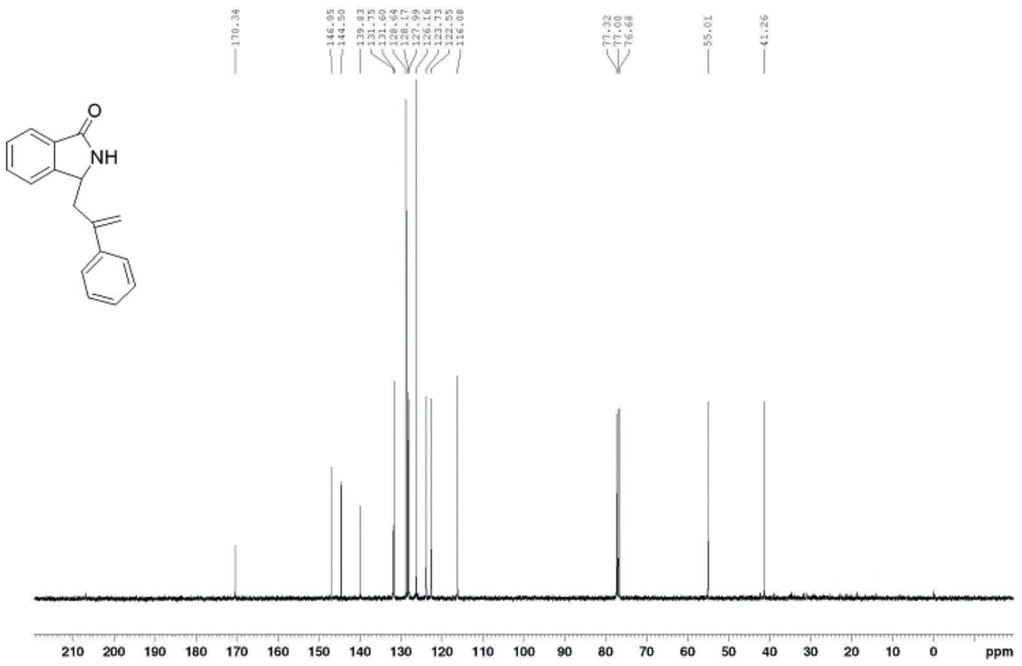

^{1}H NMR and ^{13}C NMR spectra of **3n**

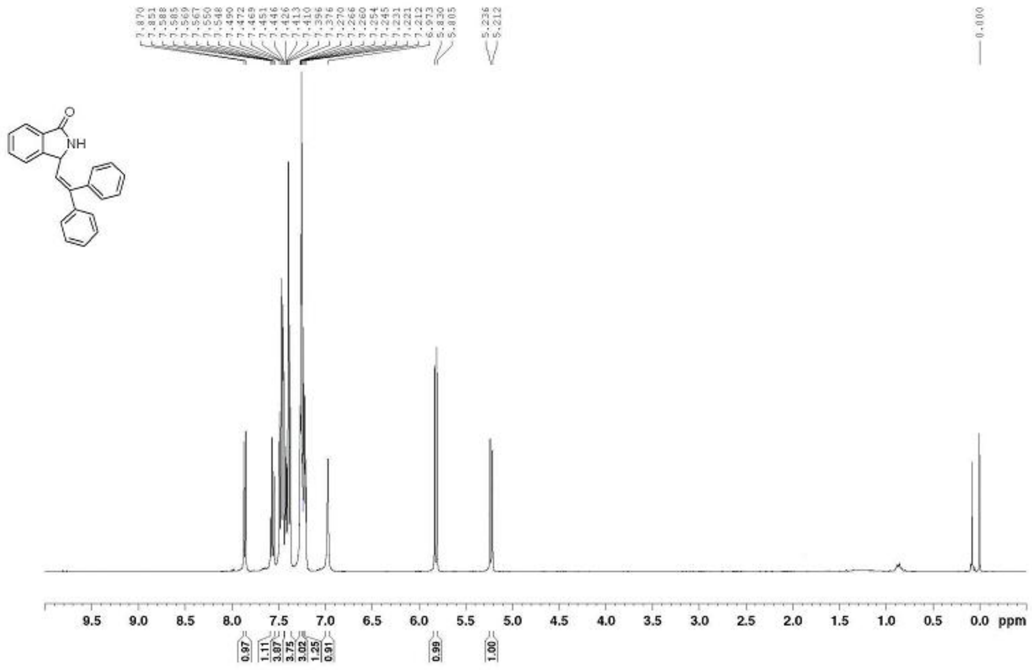

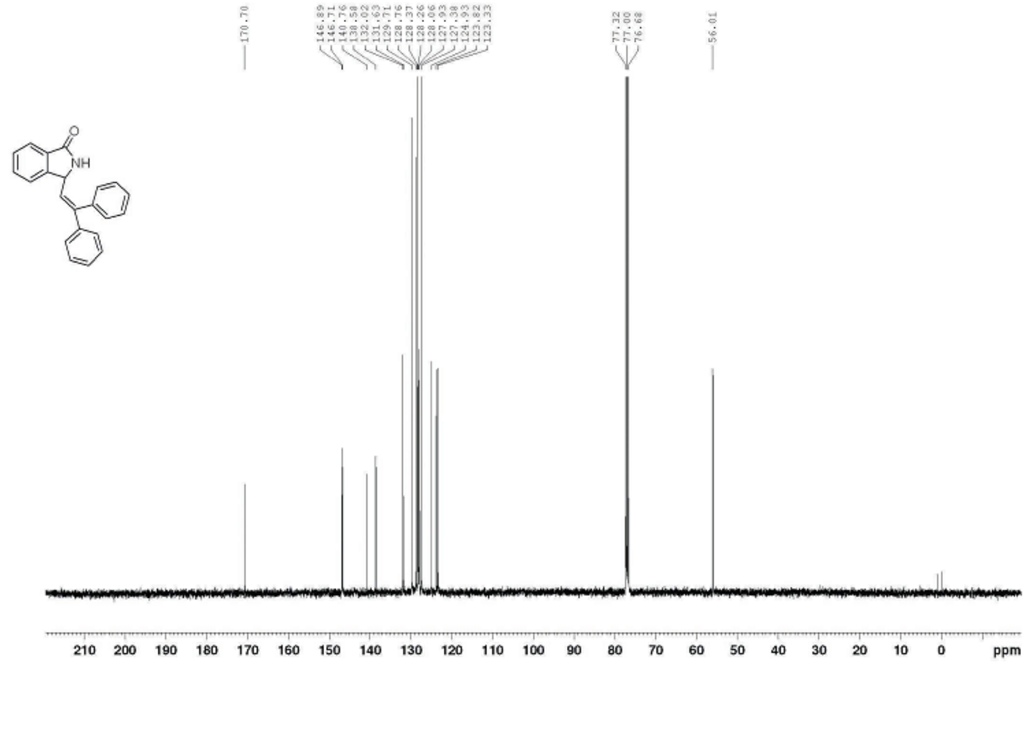

^{1}H NMR and ^{13}C NMR spectra of **3o**

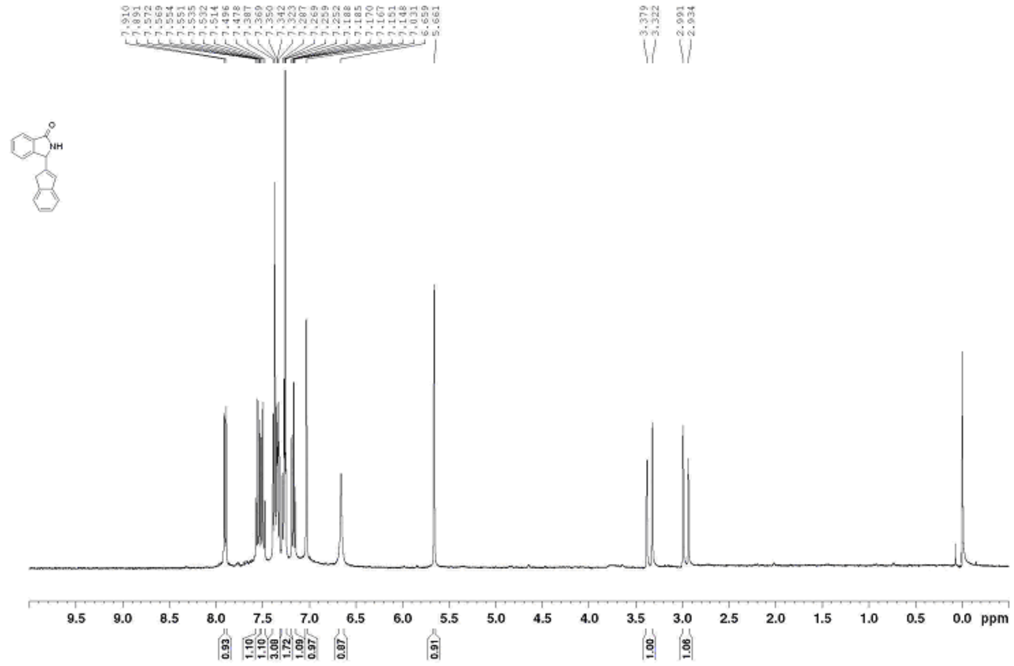

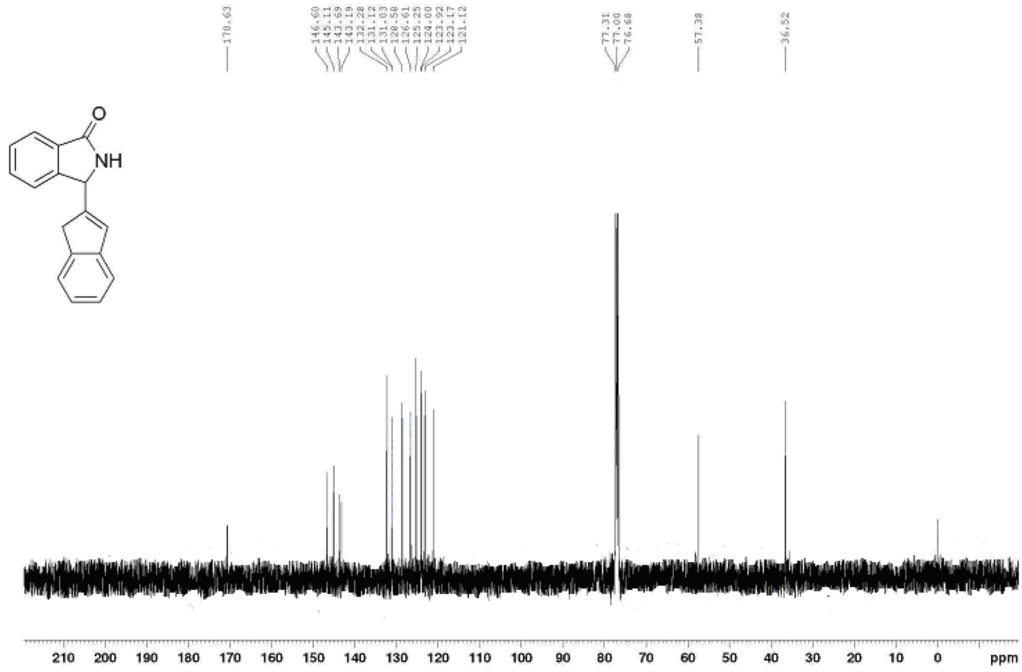

^{1}H NMR and ^{13}C NMR spectra of **6a**

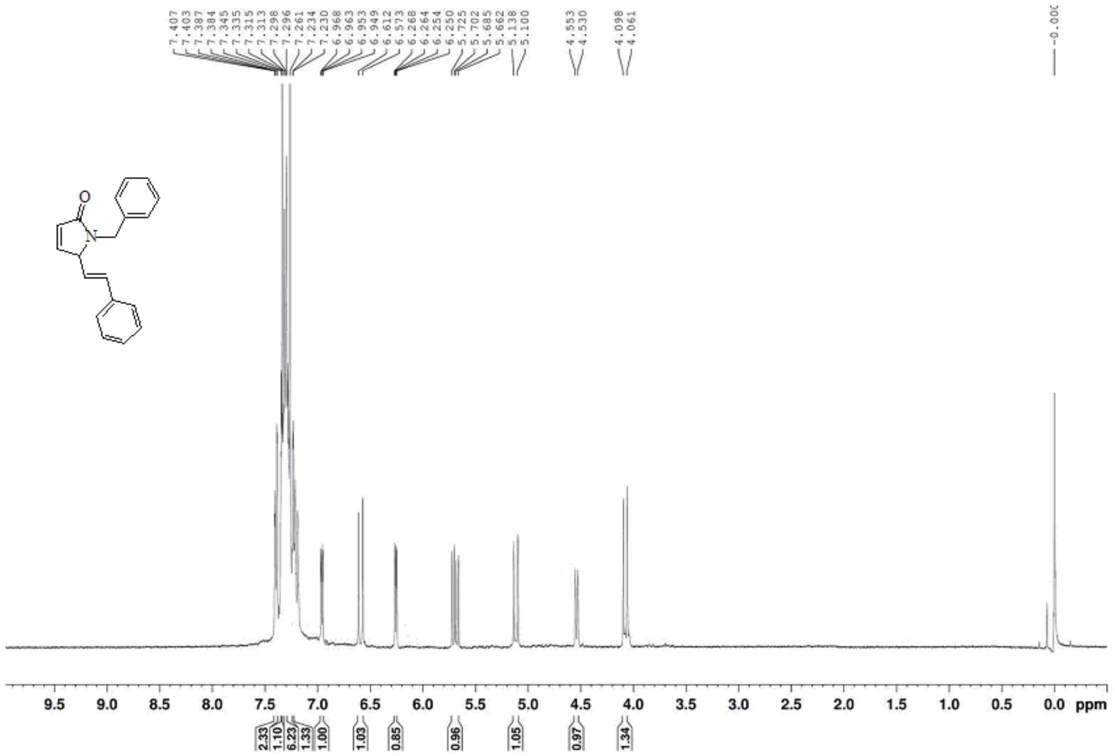

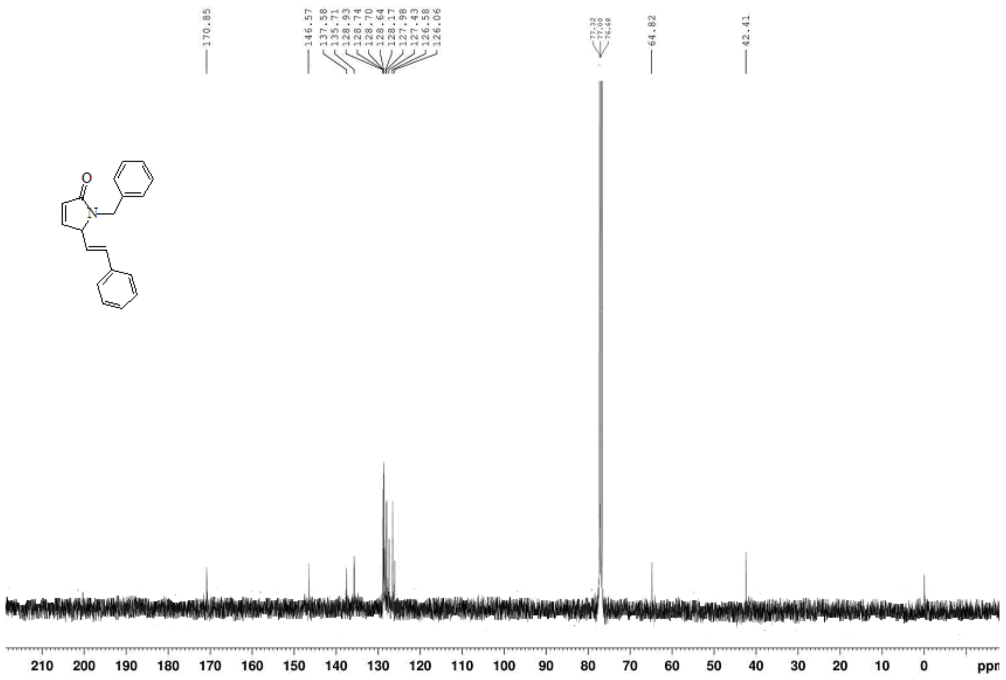

^{1}H NMR and ^{13}C NMR spectra of **7a**

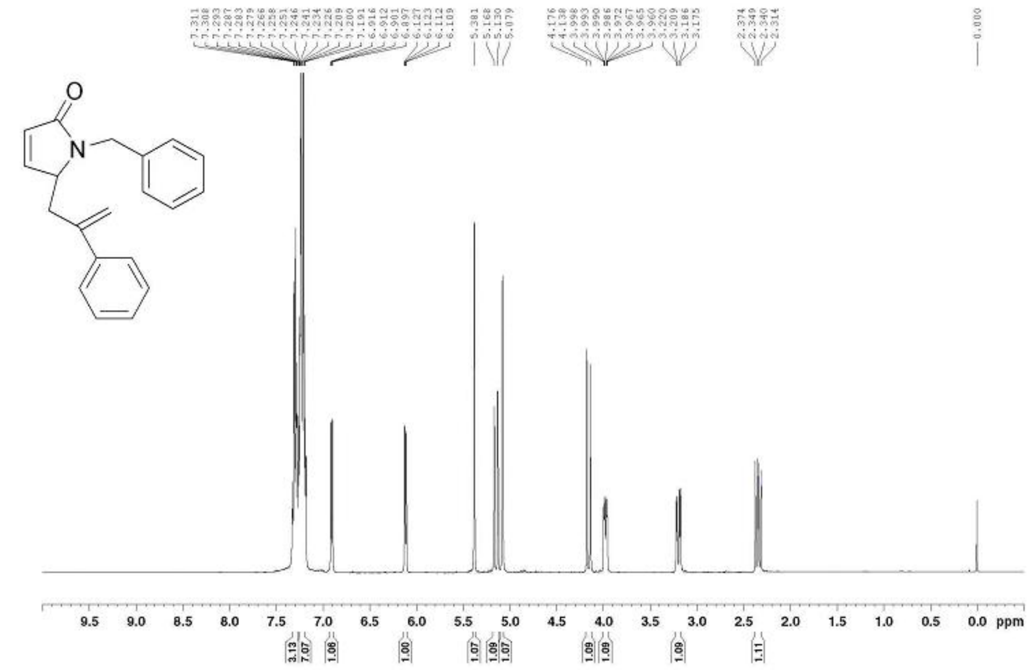

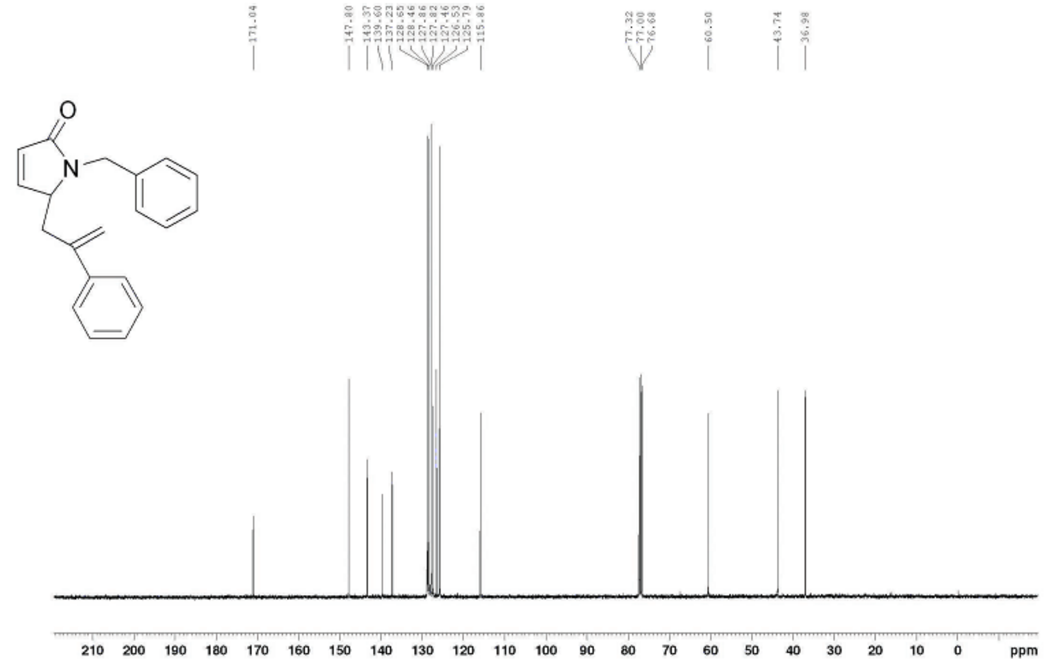

^{1}H NMR and ^{13}C NMR spectra of **7b**

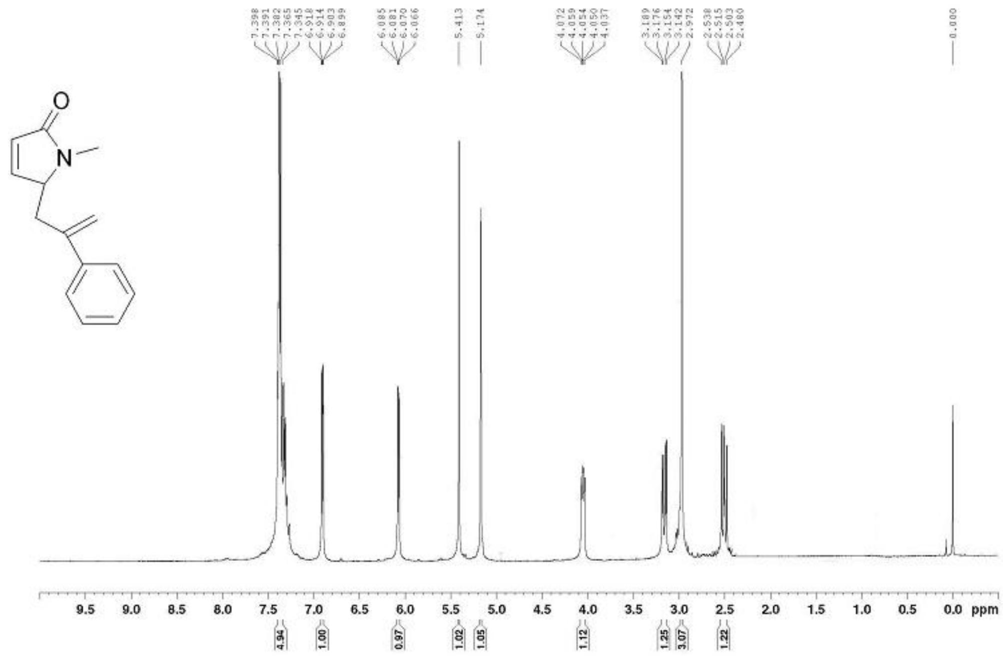

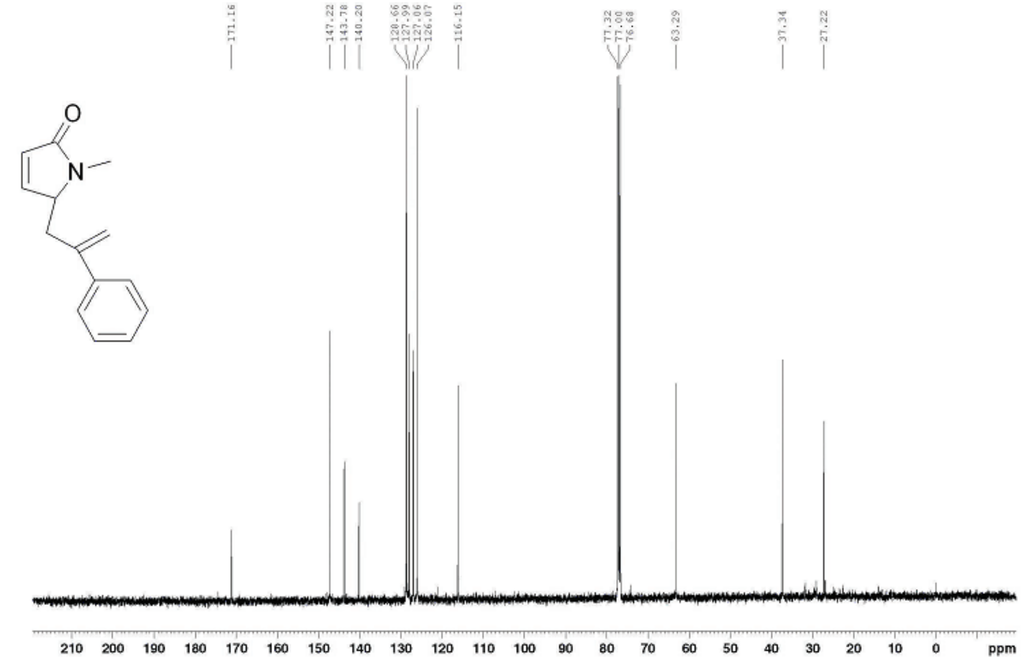

^{1}H NMR and ^{13}C NMR spectra of **6b**

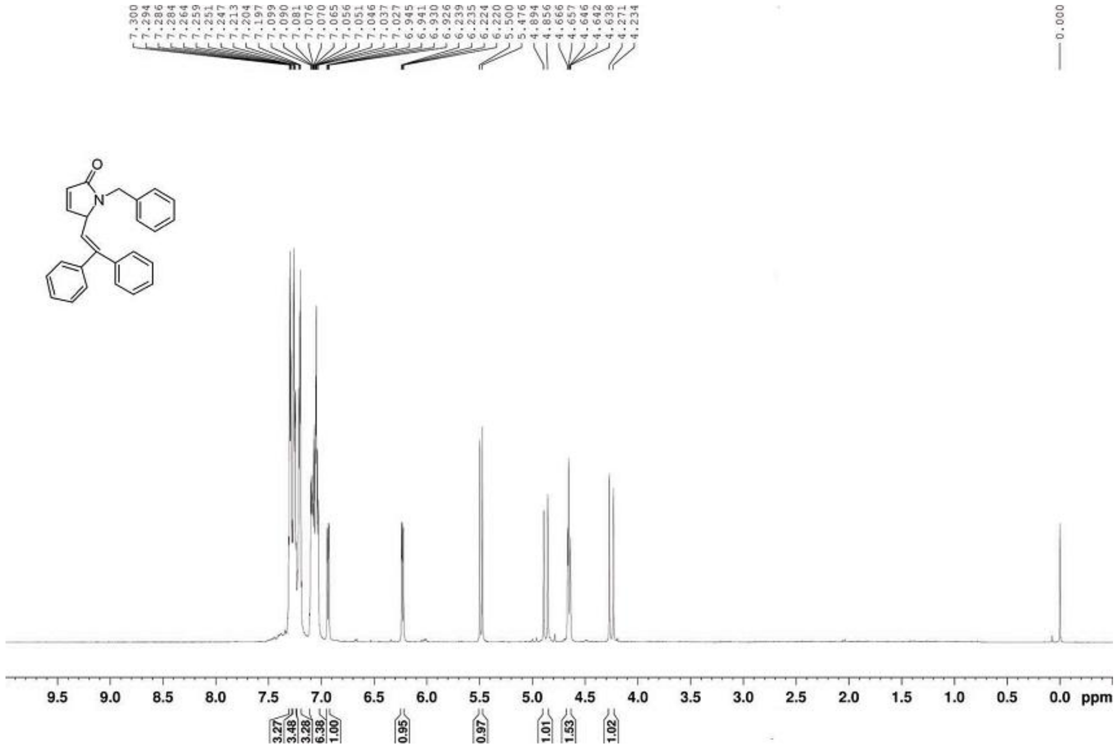

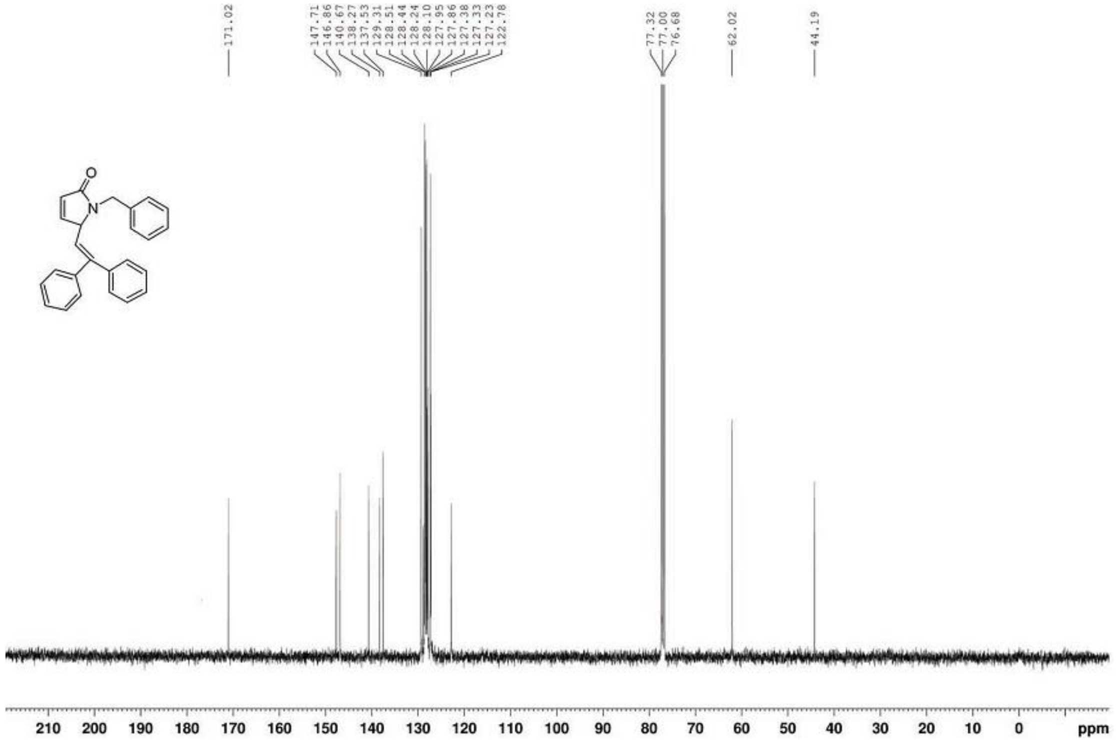

^{1}H NMR and ^{13}C NMR spectra of **6c**

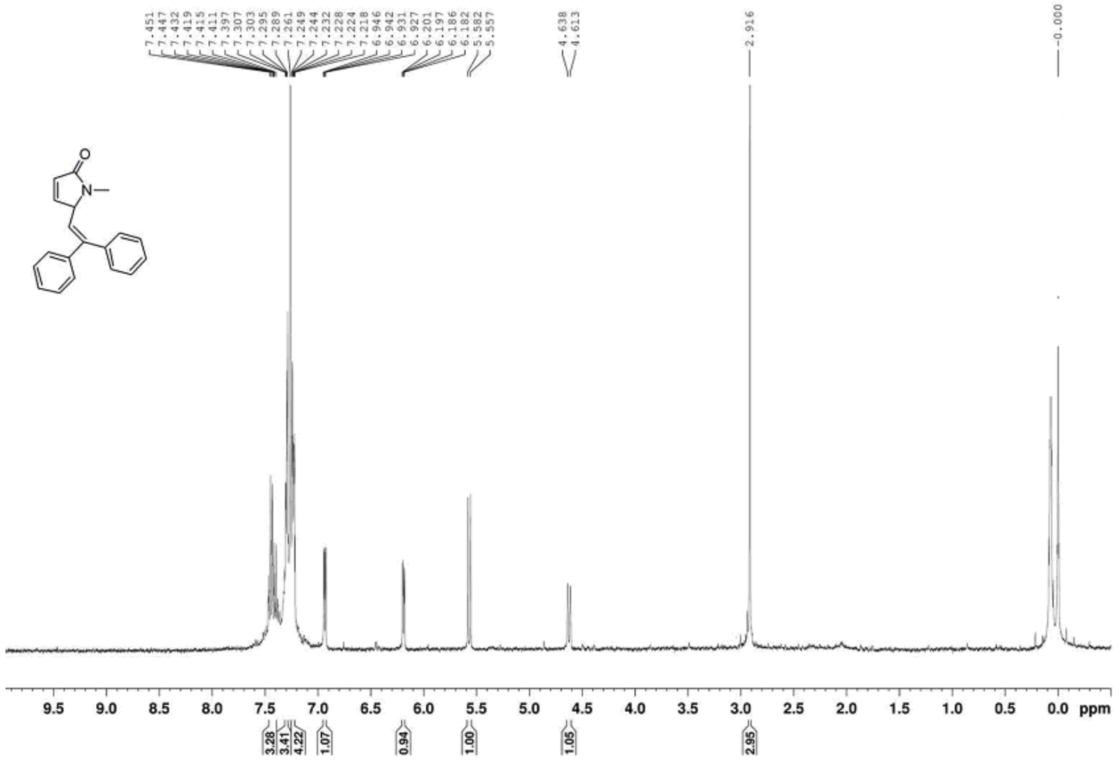

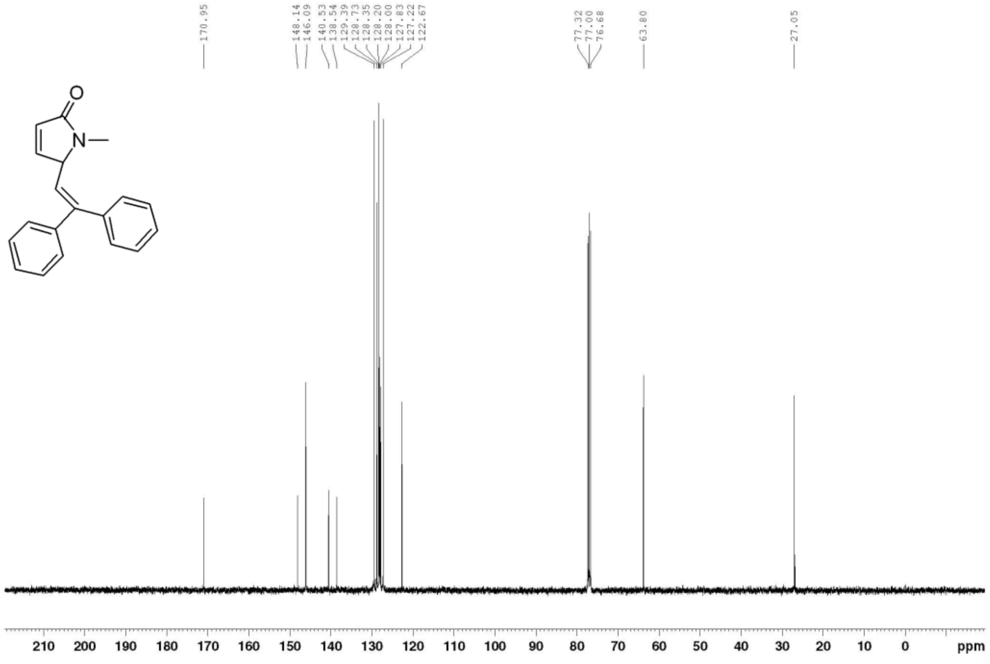

Supplement: File 1 — Characterization data of the title compounds, 1H NMR and 13C NMR spectra. [file Beilstein_J_Org_Chem-08-192-s001.pdf]
